# NOTCH4^ΔL12_16^ sensitizes lung adenocarcinomas to EGFR-TKIs through transcriptional down-regulation of HES1

**DOI:** 10.1038/s41467-023-38833-7

**Published:** 2023-06-02

**Authors:** Bin Zhang, Shaowei Dong, Jian Wang, Tuxiong Huang, Pan Zhao, Jing Xu, Dongcheng Liu, Li Fu, Lingwei Wang, Guangsuo Wang, Chang Zou

**Affiliations:** 1 Department of Respiratory and Critical Care Medicine, The First Affiliated Hospital, Southern University of Science and Technology, Shenzhen, Guangdong PR China; 2 Department of Hematology and Oncology, Shenzhen Children’s Hospital, Shenzhen, Guangdong PR China; 3 Department of Pharmacology and International Cancer Center, Shenzhen University Medical School, Shenzhen, Guangdong PR China; 4 School of Medicine, Life and Health Sciences, The Chinese University of Hong Kong (Shenzhen), Shenzhen, Guangdong PR China

**Keywords:** Non-small-cell lung cancer, Cancer therapeutic resistance, Non-small-cell lung cancer

## Abstract

Resistance to epidermal growth factor tyrosine kinase inhibitors (EGFR-TKI) remains one of the major challenges in lung adenocarcinoma (LUAD) therapy. Here, we find an increased frequency of the L12_16 amino acid deletion mutation in the signal peptide region of NOTCH4 (NOTCH4^ΔL12_16^) in EGFR-TKI-sensitive patients. Functionally, exogenous induction of NOTCH4^ΔL12_16^ in EGFR-TKI -resistant LUAD cells sensitizes them to EGFR-TKIs. This process is mainly mediated by the reduction of the intracellular domain of NOTCH4 (NICD4) caused by the NOTCH4^ΔL12_16^ mutation, which results in a lower localization of NOTCH4 in the plasma membrane. Mechanistically, NICD4 transcriptionally upregulates the expression of HES1 by competitively binding to the gene promoter relative to p-STAT3. Because p-STAT3 can downregulate the expression of HES1 in EGFR-TKI-resistant LUAD cells, the reduction of NICD4 induced by NOTCH4^ΔL12_16^ mutation leads to a decrease in HES1. Moreover, inhibition of the NOTCH4-HES1 pathway using inhibitors and siRNAs abolishes the resistance of EGFR-TKI. Overall, we report that the NOTCH4^ΔL12_16^ mutation sensitizes LUAD patients to EGFR-TKIs through transcriptional down-regulation of HES1 and that targeted blockade of this signaling cohort could reverse EGFR-TKI -resistance in LUAD, providing a potential approach to overcome resistance to EGFR-TKI -therapy.

## Abstract (web summary)

Patients with lung adenocarcinoma (LUAD) patients carrying EGFR mutations are often treated with EGFR-tyrosine kinase inhibitors (EGFR-TKIs), but sensitivity to the therapy varies. Here, using 3D printed patient derived xenograft models, the authors identify a NOTCH mutation as an indicator of favourable response to EGFR-TKI in LUAD.

## Introduction

Lung cancer remains the leading cause of cancer-related deaths worldwide^1^, and among various lung cancer subtypes, lung adenocarcinoma (LUAD) has been the fastest-growing one in recent years. Epidermal growth factor receptor tyrosine kinase inhibitors (EGFR-TKIs) have been widely used in the treatment of LUAD^2^ showing high responses while greatly improving the life expectancy of patients^3,4^. However, the sensitivity to EGFR-TKI therapy varies among patients, and most patients acquire resistance to EGFR-TKI after a certain period of effective treatment. Understanding the key mechanisms regulating the effectiveness of EGFR-TKI therapy will help improve the prognostic accuracy and treatment efficiency.

Next-generation sequencing (NGS) has been widely used in the identification of targetable oncogenic mutations and has been recognized as the standard for the treatment of patients with metastatic non-small cell lung cancer (NSCLC) in the United States^5^. Moreover, re-biopsy in recurrent patients has enabled the comparison of pretreatment and post-relapse samples to map the landscape of acquired resistance mechanisms and to develop therapies to overcome resistance^6–9^. Using this strategy, several major mechanisms of acquired resistance have been revealed in the past decade, including T790M mutations, epithelial-mesenchymal transition (EMT), and proapoptotic protein Bcl-2-like (BIM) deletion polymorphism^10,11^. Recently, it was found that osimertinib could effectively treat LUAD with T790M mutation after resistance to first- or second-generation EGFR-TKIs^3,12^, however, osimertinib treatment could also endow the tumor with resistance to this drug and this resistance mechanism remains unknown. Therefore, further studies are required to deepen our understanding of the molecular mechanisms that regulate the effectiveness of EGFR-TKI therapy.

The mammalian NOTCH family consists of four members, NOTCH1-4. Upon ligand binding, NOTCH signaling is activated, resulting in the production of the NOTCH intracellular domain (NICD), and the translocation of NICD into the nucleus further induces the transcription of downstream target genes^13^. The NOTCH signaling pathway plays an important regulatory role in a variety of tumor activities^14^. NOTCH1 activating mutations have been found in correlation with higher tumor grade of Kras-driven NSCLC. Overexpressed NOTCH2 and NOTCH3 have also been reported in association with cancer stem cell (CSC) maintenance, patient survival, and disease occurrence in NSCLC^15,16^. A high mutational proportion of NOTCH4 has also been found^17^ and is associated with a better prognosis in NSCLC^18^. However, the role of NOTCH4 mutations in the development and EGFR-TKI treatment for LUAD remains unclear.

Due to better preservation of patient tumor characteristics, Patient-derived xenograft (PDX) models are more suitable than cell line‐derived xenograft (CDX) models in the exploration of tumor progression and drug resistance mechanisms^13^. Although PDX models have been successfully established for many types of cancers including LUAD^19–22^, this construction process usually takes a lot of time, and hence it is necessary to improve this method for shortening the construction time.

In this study, we construct serially improved LUAD PDX models using a method assisted by 3D bio-printing and use these established PDX models to establish drug-resistant LUAD models for different EGFR-TKIs and compare expression profiles between parental and drug-resistant tumors samples. We find enhanced levels of a deletion mutation, NOTCH4^ΔL12_16^, in both parental tumors and original PDXs that are sensitive to EGFR-TKIs, and further demonstrate that this NOTCH4 mutation could sensitize EGFR-driven LUAD tumors to EGFR- TKIs via an intracellular NOTCH4 (NICD4)- and a HES1 dependent mechanisms.

## Results

### NOTCH4^ΔL12_16^ positively correlates with favorable efficacy of EGFR-TKIs in patients with lung adenocarcinoma

**Fig. 1:**
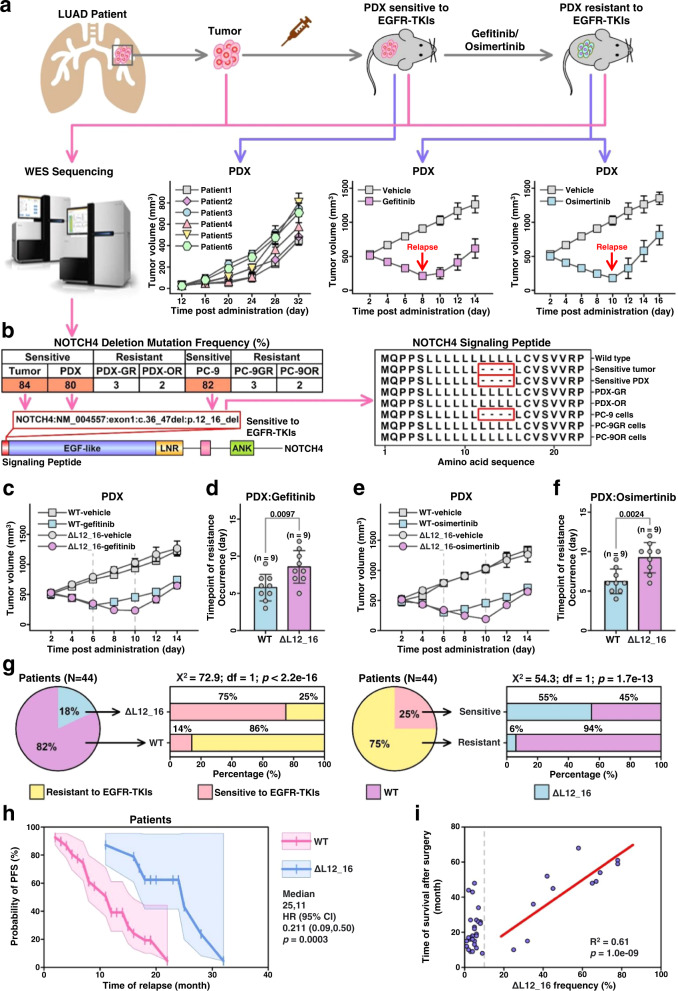
A NOTCH4ΔL12_16 mutation increases in tumors sensitive to EGFR-TKIs. **a** Schematized histories and tumor growth curves of PDX models. (*n* = 6 PDXs). **b** The frequency of NOTCH4 deletion mutations (left) and the amino acid sequences in NOTCH4 signaling peptides, red crops mean deletion sequences. **c** and **e** The tumor growth curve of PDX models. (*n* = 3 PDXs). **d** and **f** Total time of drug resistance occurrence. **g** The proportions of LUAD patients with or without NOTCH4^ΔL12_16^ mutation in sensitive or resistant patients. **h** The probability of PFS in LUAD patients with or without NOTCH4^ΔL12_16^ mutations, n = 44. **i** The correlation between NOTCH4^ΔL12_16^ frequencies and the time of survival after surgery. mean ± SD. An unpaired two-sided student t-test was performed in Fig. 1d, e. A two-sided pearson correlation analysis was performed in Fig. 1i. PDX, patient-derived xenograft; WES, whole exome sequencing. GR, gefitinib resistant; OR, osimertinib resistant; PFS, progression-free survival. Source data are provided as a Source Data file.

PDX models for EGFR-driven LUAD tumors from six patients were successfully fabricated using 3D bioprinting technology (Fig. 1a; Supplementary Fig. 1a), and the primary PDX tumors showed similar morphology, immunophenotype, and gene mutation profiles to the corresponding patient tumors (Supplementary Fig. 1b–d). After induction of TKIs, PDX tumors became resistant to gefitinib, afatinib, and osimertinib separately (Supplementary Fig. 1e). All PDX tumors derived from six patients were sensitive to these two EGFR TKIs at baseline, and drug-resistant tumors were achieved in the different PDX models after 8-12 days of treatment (Fig. 1a). To identify potential DNA alterations that might be involved in resistance to EGFR TKIs, whole-exome sequencing (WES) was performed on the DNA pools containing gefitinib- or osimertinib-resistant PDX tumors, the corresponding patient tumors, and the original PDXs that were sensitive to these drugs. DNA from the human EGFR-driven LUAD PC −9/ PC −9GR/ PC −9OR cell line (EGFR T790M) was also included in this study. The overall mutation burden and distribution patterns across the genome were similar in the paired tumors and cell lines. However, a missense mutation in NOTCH4, resulting in the deletion of four Lucien amino acids in the signaling peptide region (NOTCH4^ΔL12_16^), was found to be dramatically more prevalent in EGFR-TKI sensitive tumors/cells than in these resistant ones (Fig. 1b; Supplementary Table 1). Further investigation by polymerization chain reaction (PCR) confirmed the presence of this mutation in the tumors of three patients and the disappearance of these mutations in the corresponding EGFR-TKI-resistant tumors (Supplementary Fig. 1f). Interestingly, the overall time to the emergence of resistance in the PDX tumors derived from patient tumors with NOTCH4^ΔL12_16^ mutation (PDX-NOTCH4mut) was significantly longer than in the PDX tumors derived from patient tumors without NOTCH4^ΔL12_16^ mutation (PDX-NOTCH4wt) (Figs. 1c–f), suggesting that the NOTCH4^ΔL12_16^ mutation may sensitize LUAD tumors to treatment with gefitinib or osimertinib.

We further detected NOTCH4^ΔL12_16^ in the DNA samples of 44 EGFR-driven LUAD patient tumors, and found 18% of the samples harbored this mutation, and among these patients (18%), 75% of them remained sensitive to gefitinib or osimertinib therapy 6 months after treatment, which is significantly higher than 14% of patients without this mutation; we found 25% of the 44 EGFR-driven individuals were sensitive to gefitinib or osimertinib at 6 months after treatment, and among these patients (25%), 55% of them harbored the NOTCH4^ΔL12_16^ mutation, which is significantly higher than 6% of patients that are resistant to gefitinib or osimertinib (Fig. 1g; Table 1). In addition, patients with NOTCH4^ΔL12_16^ showed a higher probability of progression-free survival (PFS) (Fig. 1h). The frequency of NOTCH4^ΔL12_16^ is positively correlated with the time of survival after surgery (Fig. 1i). Overall, these data suggest that NOTCH4^ΔL12_16^ mutation is positively correlated with the sensitivity of EGFR-driven LUAD tumors to EGFR TKIs. Moreover, patients with NOTCH4^ΔL12_16^ showed a higher probability of progression-free survival (PFS) (Fig. 1h). The frequency of NOTCH4^ΔL12_16^ is positively correlated with the time of survival after surgery (Fig. 1i). Altogether, these data indicate that the NOTCH4^ΔL12_16^ mutation is positively correlated with the sensitivity of EGFR-driven LUAD tumors to EGFR-TKIs.

**Table 1:** Patient characteristics and responses to EGFR-TKIs

		Total	Response to EGFR-TKIs (6 months)	
			PR + SD	PD
		*N* = 44	*N* = 33	*N* = 11
Age (years)	Medium	45	48	52
	Range	32–78	32–78	41–78
Gender	Male	22	15	7
	Female	22	18	4
Histology	Adenoca.	44	33	11
	Other	0	0	0
Stage	III	21	13	8
	IV	23	20	3
EGFR status	WT	0	0	0
	Mutant	44	33	11
NOTCH4 status	WT	36	5	31
	ΔL12_16	8	6	2

*PR* partial response, *SD* stable disease, *PD* progressive disease, *Adenoca* lung adenocarcinoma.

### Exogenous NOTCH4^ΔL12_16^ increases the sensitivity of LUAD to EGFR-TKIs

**Fig. 2:**
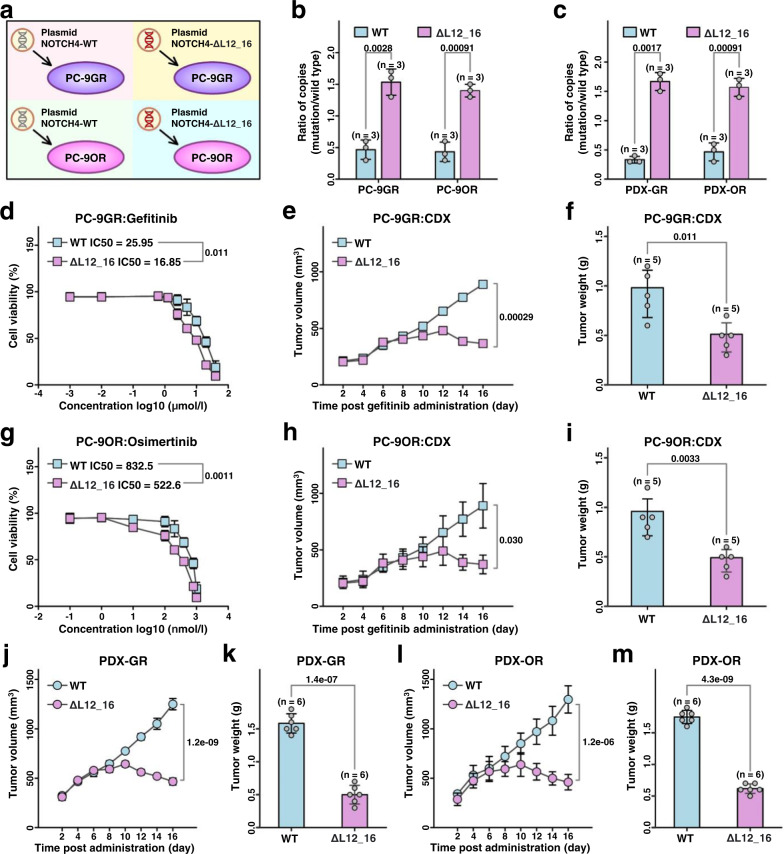
Exogenous NOTCH4ΔL12_16 transfection sensitizes LUAD to EGFR-TKIs. **a** The diagram of NOTCH4 plasmids transfection into PC-9GR/PC-9OR. **b**, **c** The ratio of mutation after NOTCH4 plasmids transfection into PC-9GR/PC-9OR or tumor cells of PDX-GR/PDX-OR compared with wild type. The data shown are representative of at least 3 independent repeats. (*n* = 3 PDXs). **d** and **g** The cell viability after NOTCH4 plasmids transfection into PC-9GR/PC-9OR (*n* = 3 biologically independent experiments). **e** and **h** The tumor growth curves of CDX models. (*n* = 5 CDXs). **f** and **i** Tumor weight of CDX models. (*n* = 5 CDXs). (**j**, **l**) The tumor growth curve of PDX models. (*n* = 6 PDXs). **k** and **m** Tumor weight of PDX models. (*n* = 6 PDXs) mean ± SD. An unpaired two-sided student t-test was performed in Figs. 2b–m. CDX, cell-derived xenograft; PDX, patient-derived xenograft. Source data are provided as a Source Data file.

To further investigate the role of the NOTCH4^ΔL12_16^ mutation in enhancing the sensitivity of EGFR-driven LUAD tumors to EGFR TKIs, plasmids of NOTCH4 with the ΔL12_16 mutation (NOTCH4mut) were transfected into PC-9/ PC-9GR/ PC-9OR cells or PDX-GR/PDX-OR tumors (Figs. 2a–c, Supplementary Fig. 2a–c) were transfected and verified (Supplementary Fig. 9a,b), and their effects on the sensitivity of EGFR-TKI treatments were measured. NOTCH4^ΔL12_16^ transfection decreased the half-maximal inhibitory concentration (IC50) of gefitinib (Fig. 2d) and osimertinib (Fig. 2g) in PC-9GR and PC-9OR cells, respectively. The in vivo sensitivity of these transfected cells to EGFR-TKIs was also measured. Consistent with the in vitro study, treatment with gefitinib in NOTCH4^ΔL12_16^ PC-9GR tumors showed a greater inhibitory effect than in NOTCH4wt PC-9GR tumors (Fig. 2e, f). Similarly, NOTCH4^ΔL12_16^ transfection significantly increased in vivo sensitivity to osimertinib in PC-9GR cells compared with NOTCH4wt transfection (Fig. 2h, i). Very similar results were found in EGFR-TKI-resistant PDX models (Fig. 2j–m). Intra-tumoral injection of NOTCH4^ΔL12_16^ plasmids instead of NOTCH4wt plasmids in PDX-GR tumors or PDX-OR tumors sensitized them to gefitinib and osimertinib separately. We also identified the NOTCH4^ΔL12_16^ protein and introduced the mutation along with null alleles using CRISPR-Cas9 genome editing in PC9-GR and PC-9OR. These isogenic cell lines allow comparison of mutant (NOTCH4^ΔL12_16^ + /−), wild-type (NOTCH4^ΔL12_16^ + /+), and null alleles (NOTCH4^ΔL12_16^−/−) expressed from the endogenous locus, thus preserving the full complexity of NOTCH4^ΔL12_16^ feedback mechanisms, and similar results were obtained (Supplementary Fig. 6). Taken together, these results suggest that the NOTCH4^ΔL12_16^ mutation increases the sensitivity of LUAD to EGFR TKIs.

### NOTCH4^ΔL12_16^ sensitizes LUAD to EGFR-TKIs by reducing intracellular NOTCH4 (NICD4)

**Fig. 3:**
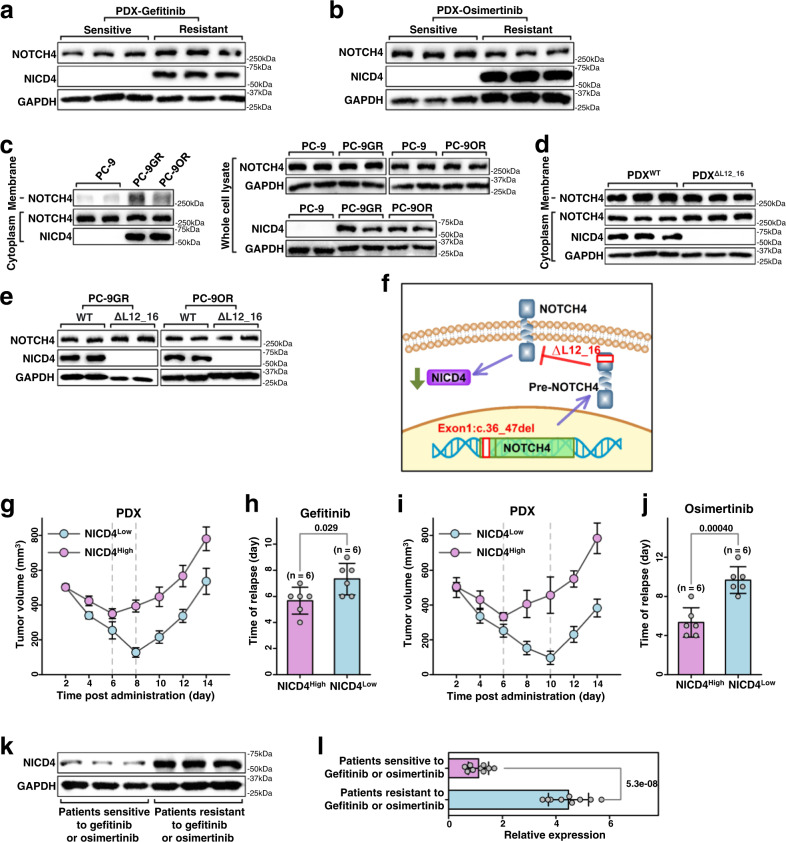
The sensitivity of lung adenocarcinoma to EGFR-TKIs correlated with NICD4 expression. **a**, **b** Western blot analysis of protein extracts from isolated tumor cells of PDX models resistant or sensitive to gefitinib or osimertinib. **c**, **d** Western blot analysis of protein extracts from isolated tumor cells of PC-9/PC-9GR/PC-9OR or PDX models with or without NOTCH4^ΔL12_16^ mutations. **e** Western blot analysis of protein extracts from PC-9GR/PC-9OR cells transfected with NOTCH4 plasmids. **f** The diagram of how NOTCH4^ΔL12_16^ mutations affect NICD4 expression. **g** and **i** The tumor growth curve of PDX models of high expression NICD4 compared with low expression NICD4, dotted line means occurrence of relapse. (*n* = 3 PDX). **h** and **j** The total time of relapse occurrence of PDX models of NICD4 high expression compared with low expression. (*n* = 6 PDXs). **k**, **l** Western blot analysis of protein extracts from isolated tumor cells of patients sensitive or resistant to gefitinib or osimertinib. (*n* = 18 Patients) mean ± SD. An unpaired two-sided student t-test was performed in Fig. 3h, j, and l. Three biologically independent experiments were performed for Fig. 3a–e. NICD4, NOTCH4 intracellular domain fragment. Source data are provided as a Source Data file.

We next investigated the mechanisms by which the NOTCH4^ΔL12_16^ mutation increases the sensitivity of LUAD to EGFR TKIs. As reported, the NOTCH4^ΔL12_16^ mutation occurred in signal peptides, which are short (5-30 amino acids long) peptide chains that direct the transfer of newly synthesized proteins into the secretory pathway. Moreover, the full-length NOTCH4 (NOTCH4- FL) would release intracellular NOTCH4 (NICD4) only when it is at the plasma membrane, further activating downstream NOTCH4 signaling^23^. Therefore, the NOTCH4^ΔL12_16^ mutation could inhibit the translocation of NOTCH4 to the plasma membrane, decreasing NICD4 levels and inactivating NOTCH4 signaling. As shown in Fig. 3a, b, EGFR-TKI -sensitive primary LUAD tumors had lower NICD4 levels compared with EGFR-TKI -resistant PDX tumors. Accordingly, there was less membrane-derived NOTCH4- FL and cytosol-derived NICD4 in PC-9 cells compared with PC-9GR and PC-9OR cells (Fig. 3c and supplemental Fig. 3c). Moreover, transfection of exogenous NOTCH4^ΔL12_16^ into PDX tumors or PC-9 cells decreased NOTCH4- FL levels in the membrane and NICD4 levels in the cytosol, whereas NOTCH4- FL levels in the cytoplasm were increased (Fig. 3d–f). To further investigate the relationship between NICD4 levels and EGFR-TKI sensitivity in LUAD, we divided nine EGFR-driven PDX tumors into two groups: PDXs with high NICD4 levels and PDXs with low NICD4 levels (supplementary Fig. 3a). Interestingly, PDX tumors with lower NICD4 expression were more sensitive to gefitinib or osimertinib than those with higher NICD4 levels (Fig. 3g–j and Supplementary Fig. 3b). Moreover, NICD4 expression in EGFR-driven LUAD patient tumors was significantly lower in patients sensitive to EGFR-TKI therapy than in resistant patients (Fig. 3k, l). These data suggest that NICD4 protein levels are inversely correlated with EGFR-TKI sensitivity in EGFR-driven LUAD tumors.

**Fig. 4:**
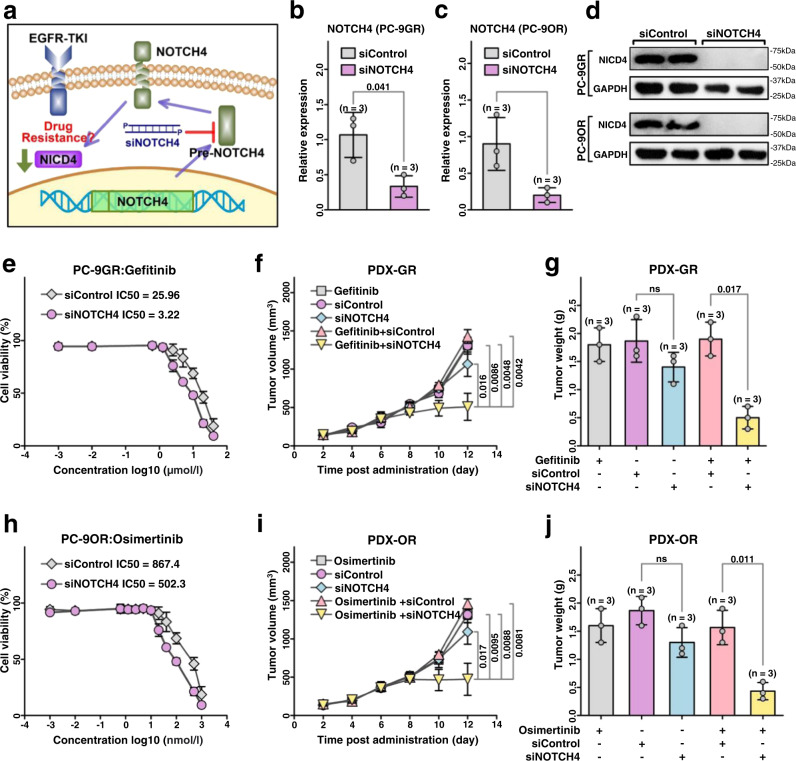
Silence of NOTCH4 via siNOTCH4 sensitizes LUAD tumors to EGFR-TKIs. **a** The diagram of siNOTCH4 inhibiting NICD4 expression. **b**, **c** The relative expression of NOTCH4 after siNOTCH4 treatment in PC-9GR/PC-9OR. (*n* = 3 biologically independent experiments). **d** Western blot analysis of NICD4 in PC-9GR/PC-9OR with siNOTCH4 treatment. **e** and **h** The cell viability after siNOTCH4 treatment in PC-9GR/PC-9OR. (*n* = 3 biologically independent experiments). **f** and **i** The tumor growth curve of PDX models with siNOTCH4 or gefitinib/osimertinib treatments. (*n* = 3 PDXs). **g** and **j** The tumor weight of PDX models with siNOTCH4 or gefitinib/osimertinib treatments. (*n* = 3 PDXs) mean ± SD. ns, not significant; An unpaired two-sided student t-test was performed in Fig. 4b, c, f, g, i, and j. NICD4, NOTCH4 intracellular domain fragment. Source data are provided as a Source Data file.

To investigate the role of NICD4 downregulation in increasing the sensitivity of EGFR-driven LUAD tumors to EGFR-TKIs, we decreased NICD4 levels in PC-9GR and PC-9OR cells using a pool of siRNAs targeting NOTCH4 mRNA (siNOTCH4) (Fig. 4a) and further verified by real-time PCR (supplementary Fig. 7a, b). The expression of NOTCH4 mRNA was significantly decreased in siNOTCH4-treated cells PC-9GR and PC-9OR compared with siControl-treated cells (Fig. 4b, c). Importantly, the expression of NICD4 was inhibited in siNOTCH4-treated cells compared with siControl-treated cells (Fig. 4d). The IC50 values of both PC −9GR and PC −9OR cells decreased significantly after siNOTCH4 treatment (Fig. 4e, h). Moreover, siNOTCH4 treatment synergistically enhanced the inhibitory effects of gefitinib (Fig. 4f, g) and osimertinib (Fig. 4i, j) in EGFR-driven LUAD PDX tumors. Of note, treatment with siNOTCH4 alone did not significantly suppress tumor growth (Fig. 4f, g, i, and j). These data indicate that depletion of NICD4 may sensitize LUAD tumors to EGFR TKIs.

### Reduction of NICD4 sensitizes LUAD to EGFR-TKIs in a HES1-dependent manner

**Fig. 5:**
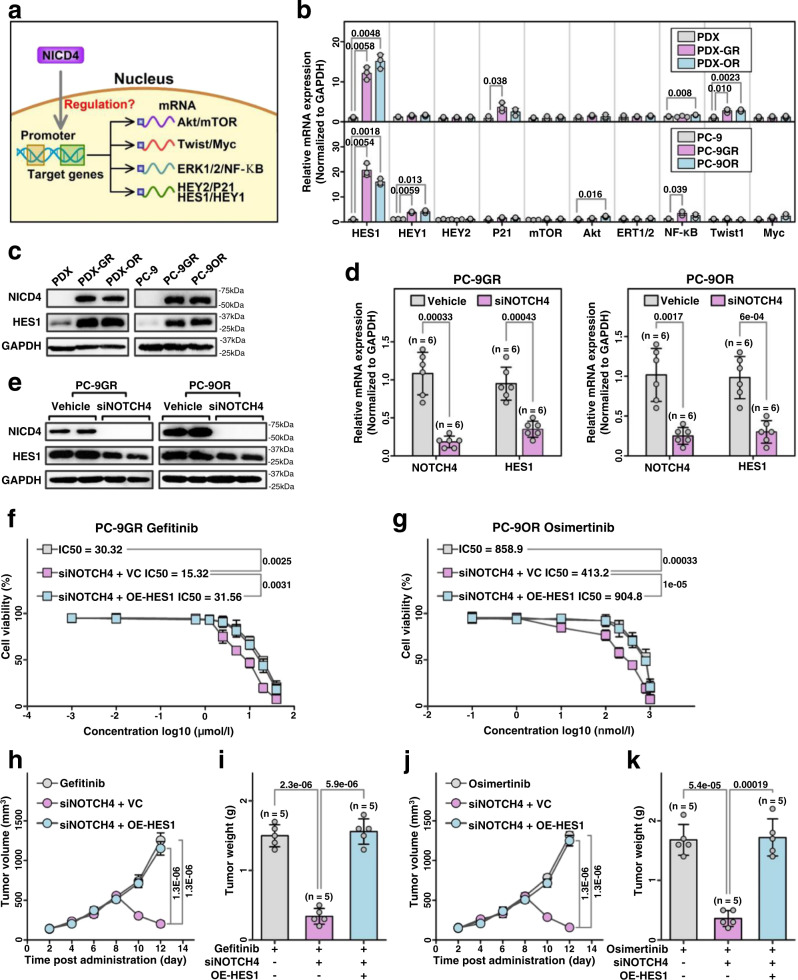
Reduction of intracellular NOTCH4 (NICD4) sensitized EGFR-TKIs mediated by HES1. **a** The diagram of potential downstream molecules of NOTCH4. **b** The relative mRNA expressions of potential downstream of NOTCH4. (*n* = 3 biologically independent experiments). **c** Western blot analysis of tumor cells in PDX/PDX-GR/PDX-OR and PC-9/ PC-9GR /PC-9OR cells. **d** The relative mRNA expressions in PC-9GR/PC-9OR with siNOTCH4 treatment. (*n* = 6 biologically independent experiments). **e** Western blot analysis of PC-9GR/PC-9OR with siNOTCH4 treatment. **f** and **g** The cell viability after siNOTCH4 with or without OE-HES1 treatment in PC-9GR/PC-9OR. (*n* = 3 biologically independent experiments). **h** and **j** The tumor growth curve of CDX models after siNOTCH4 with or without OE-HES1 treatments. (*n* = 5 CDXs). **i** and **k** The tumor weight of CDX models after siNOTCH4 with or without OE-HES1 treatments. (*n* = 5 CDXs) mean ± SD. An unpaired two-sided student t-test was performed in Fig. 5b, d, and 5f–k. Three biologically independent experiments were performed for Fig. 5c, e. Source data are provided as a Source Data file.

NOTCH4 may be involved in the regulation of HES1/HEY1/HEY2/P21/ ERK /NF-κB, Twist/Myc, and mTOR/Akt signals (Fig. 5a and Supplementary Fig. 4). All these signals were compared between the LUAD PDX - tumor or cells sensitive to EGFR-TKIs and those resistant to EGFR-TKIs. HES1 showed the most significant increase in EGFR-TKI -resistant tumors/cells compared with EGFR-TKI sensitive tumors (Fig. 5b; Table 2). Increased levels of HES1 protein were also detected in EGFR-TKI -resistant tumors/cells and not in EGFR-TKI -sensitive tumors or cells (Fig. 5c). Moreover, knockdown of NOTCH4 using siNOTCH4 reduced mRNA (Fig. 5d) and protein (Fig. 5e) expression of HES1 in PC-9GR or PC-9OR cells, suggesting that HES1 expression is positively regulated by NOTCH4. To investigate the role of HES1 signaling in NICD4 reduction that sensitizes LUAD tumors to EGFR TKIs, HES1 was overexpressed in PC-9GR or PC-9OR cells in which NOTCH4 was knocked down with a pool of siRNAs targeting NOTCH4 mRNA. NICD4 reduction induced by siNOTCH4 treatment decreased the IC50 of gefitinib and osimertinib in PC-9GR or PC-9OR cells, respectively, and the change in IC50 was reversed when HES1 was overexpressed (Fig. 5f, g). Moreover, induction of HES1 overexpression overcame the increase in sensitivity to gefitinib (Fig. 5h, i) or osimertinib (Fig. 5j, k) induced by siNOTCH4 treatment in LUAD PDX tumors. Taken together, these results suggest that the reduction of intracellular NOTCH4 (NICD4) sensitizes EGFR TKIs in LUAD via HES1 inhibition.

**Table. 2:** Primers for qPCR

Genes		Sequences (5’−3’)
NOTCH4	Forward	TTCCACTGTCCTCCTGCCAGAA
	Reverse	TGGCACAGGCTGCCTTGGAATC
HES1	Forward	GGAAATGACAGTGAAGCACCTCC
	Reverse	GAAGCGGGTCACCTCGTTCATG
HEY1	Forward	TGTCTGAGCTGAGAAGGCTGGT
	Reverse	TTCAGGTGATCCACGGTCATCTG
HEY2	Forward	TGAGAAGACTTGTGCCAACTGCT
	Reverse	CCCTGTTGCCTGAAGCATCTTC
P21	Forward	AGGTGGACCTGGAGACTCTCAG
	Reverse	TCCTCTTGGAGAAGATCAGCCG
mTOR	Forward	AGCATCGGATGCTTAGGAGTGG
	Reverse	CAGCCAGTCATCTTTGGAGACC
Akt	Forward	TGGACTACCTGCACTCGGAGAA
	Reverse	GTGCCGCAAAAGGTCTTCATGG
ERT1/2	Forward	ACACCAACCTCTCGTACATCGG
	Reverse	TGGCAGTAGGTCTGGTGCTCAA
NF-κB	Forward	GCAGCACTACTTCTTGACCACC
	Reverse	TCTGCTCCTGAGCATTGACGTC
Twist1	Forward	GCCAGGTACATCGACTTCCTCT
	Reverse	TCCATCCTCCAGACCGAGAAGG
Myc	Forward	CCTGGTGCTCCATGAGGAGAC
	Reverse	CAGACTCTGACCTTTTGCCAGG

### NICD4 transactivates HES1 in LUAD by competitive binding to its gene promoter relative to p-STAT3

**Fig. 6:**
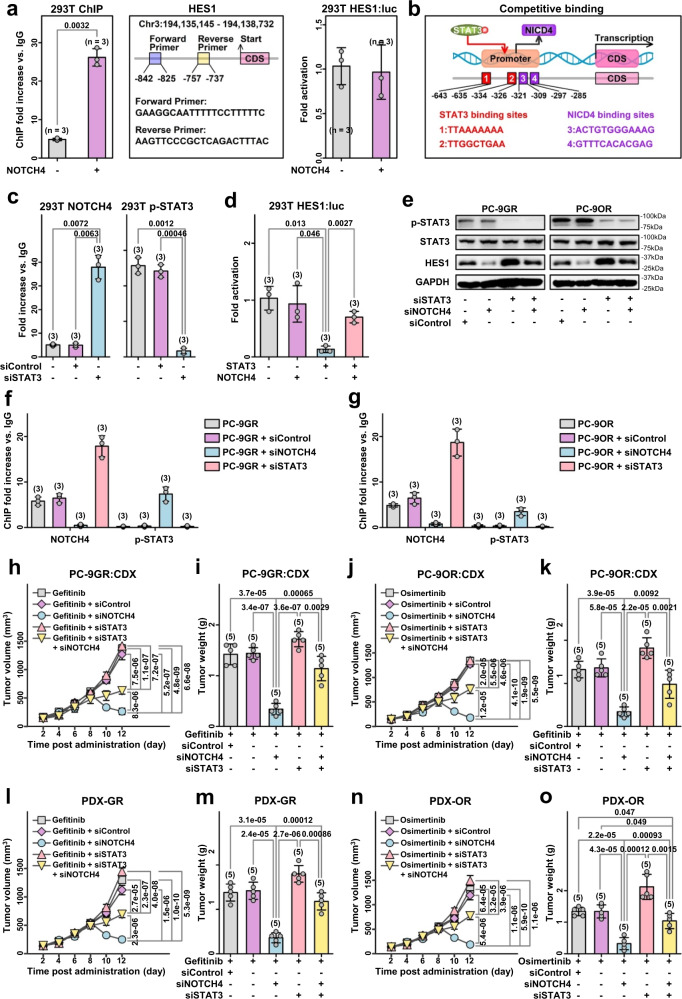
Intracellular NOTCH4 (NICD4) transactivates HES1 by competitive binding with its gene promoter over p-STAT3. **a** ChIP analysis of NOTCH4 binding to the HES1 promoter in 293 T cells and luciferase analysis of NOTCH4 to HES1. (*n* = 3 biologically independent experiments). **b** The predicted binding sites of STAT3 and NICD4 to HES1 promoter. **c** ChIP analysis of NOTCH4 and p-STAT3 binding to the HES1 promoter in 293 T cells. (*n* = 3 biologically independent experiments). **d** Luciferase analysis of NOTCH4 and STAT3 to HES1 in 293 T cells (*n* = 3 biologically independent experiments). **e** Western blot analysis in PC-9GR/PC-9OR. **f** and **g** ChIP analysis of NOTCH4 and p-STAT3 binding to the HES1 promoter in PC-9GR/PC-9OR cells treated with siNOTCH4 or siSTAT3. (*n* = 3 biologically independent experiments). **h** and **j** The tumor growth curve of CDX models with siNOTCH4, siSTAT3 or their combination treatments. (*n* = 5 CDXs). (**i** and **k**) The tumor weight of CDX models with siNOTCH4, siSTAT3 or their combination treatment. (*n* = 5 CDXs). **l** and **n** The tumor growth curve of PDX models of PC-9GR/PC-9OR with siNOTCH4, siSTAT3 or their combination treatments. (*n* = 5 PDXs). **m** and **o** The tumor weight of PDX models of PC-9GR/PC-9OR with siNOTCH4, siSTAT3 or their combination treatments. (*n* = 5 PDXs) mean ± SD. An unpaired two-sided student t-test was performed in Fig. 6a–d, and 6f–o. Three biologically independent experiments were performed for Fig. 6e. Source data are provided as a Source Data file.

To elucidate the underlying mechanism of regulation of HES1 by NICD4, we first examined the ability of NICD4 to directly transactivate HES1. Figure 6a shows that NICD4 binds to the HES1 promoter in 293 T cells but does not significantly transactivate it. Therefore, NICD4 could upregulate HES1 transcription by interfering with transcriptional suppressor binding. EGFR-TKIs could activate STAT3 signaling, and phospho-STAT3 (p-STAT3) could bind to the promoter of HES1 and repress its transcription^24^. As a binding site, we found that the consensus binding sites for p-STAT3 (i.e., TTNNNNAA)^25^ in the human HES1 and mouse gene promoters were located near RBPJ sites (i.e., where the NOTCH transcription complex binds) (Fig. 6b). Activation of STAT3 induced by gefitinib and osimertinib was also confirmed (Fig. 6e). Moreover, p-STAT3 was able to bind to the HES1 promoter and repress its transcription and expression in 293 T cells (Supplementary Fig. 5a-5b and Supplementary Info 1). We next investigated whether NICD4 could regulate the suppressive effect of p-STAT3 on HES1 transcription and expression. Using a luciferase reporter assay, we found that STAT3 could directly inactivate the HES1 promoter and, most importantly, that NICD4 expression could overcome the STAT3-induced inactivation of the HES1 promoter (Fig. 6d). Moreover, ChIP experiments in 293 T, gefitinib-treated PC - 9GR and osimertinib-treated PC - 9OR cells demonstrated that p-STAT3 can bind to the HES1 promoter and that silencing of NOTCH4 can significantly enhance the binding of p-STAT3 to HES1 (Fig. 6c, f, g and Supplementary Fig. 7d). Similarly, the binding of NOTCH4 to the HES1 promoter increased after silencing of p-STAT3 (Fig. 6f, g and Supplementary Fig. 7c). These data suggest that NICD4 transactivates HES1 by competitively binding to its gene promoter relative to p-STAT3. We further investigated the role of this competitive binding in regulating the sensitivity of LUAD tumors to EGFR TKIs. When p-STAT3 was reduced by siSTAT3 (Fig. 6e), the decrease in HES1 and the increase in drug sensitivity induced by siNOTCH4 treatment were significantly reversed in gefitinib-treated PC - 9GR cells and osimertinib-treated PC - 9OR cells (Fig. 6h–k). Moreover, siSTAT3 treatment attenuated the anti-tumor effects of siNOTCH4 treatment in EGFR-TKI-treated LUAD-PDX (Fig. 6l–o). Overall, these findings support the notion that NICD4 reduction inhibits HES1 signaling and increases EGFR-TKI sensitivity by creating opportunities for p-STAT3 binding to the HES1 promoter and repressing its transcription in LUAD.

### Blockade of NOTCH4 or HES1 by inhibitors could overcome EGFR-TKIs resistance in LUAD

**Fig. 7:**
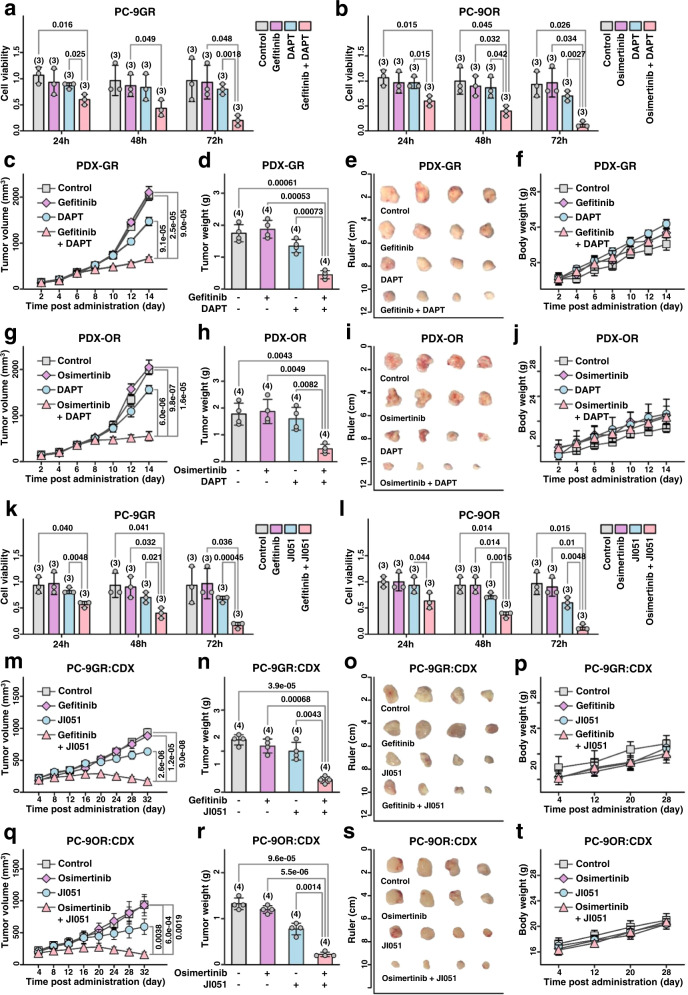
Inhibition of NOTCH4 or HES1 reverse EGFR-TKIs resistance in LUAD. **a**, **b** The cell viability with gefitinib/osimertinib, DAPT or their combination treatments in PC-9GR/PC-9OR. (*n* = 3 biologically independent experiments). **c** and **g** The tumor growth curve of PDX-GR/PDX-OR models with gefitinib/osimertinib, DAPT or their combination treatments. (*n* = 4 biologically independent experiments). **d** and **h** The tumor weight of PDX-GR/PDX-OR models with gefitinib/osimertinib, DAPT or their combination treatment. (*n* = 4 PDXs). **e** and **i** The tumor pictures of PDX-GR/PDX-OR models with gefitinib/osimertinib, DAPT or their combination treatment. **f** and **j** The body weight of PDX-GR/PDX-OR models with gefitinib/osimertinib, DAPT or their combination treatment. (*n* = 4 biologically independent experiments). (**k** and **l**) The cell viability with gefitinib/osimertinib, JI051 or their combination treatments in PC-9GR/PC-9OR. (*n* = 3 biologically independent experiments). **m** and **q** The tumor growth curve of CDX models of PC-9GR/PC-9OR with gefitinib/osimertinib, JI051 or their combination treatments. (*n* = 4 biologically independent experiments). **n** and **r** The tumor weight of CDX models of PC-9GR/PC-9OR with gefitinib/osimertinib, JI051 or their combination treatment. (*n* = 4 biologically independent experiments). **o** and **s** The tumor pictures of CDX models of PC-9GR/PC-9OR with gefitinib/osimertinib, JI051 or their combination treatment. **p** and **t** The body weight of CDX models of PC-9GR/PC-9OR with gefitinib/osimertinib, JI051 or their combination treatments. (*n* = 4 biologically independent experiments) mean ± SD. An unpaired two-sided student t-test was performed in Fig. 7a–d, g–h, k–n, and q, r. Source data are provided as a Source Data file.

Because NICD4 reduction could sensitize LUAD tumors to EGFR-TKI therapy via HES1 inhibition, we explored the possibility of overcoming EGFR-TKI resistance with NOTCH4 or HES1 inhibitors. Combined treatment with DAPT and gefitinib resulted in higher in vitro toxicity in PC -9GR cells than treatment alone (Fig. 7a). A similar role of DAPT in increasing drug sensitivity was observed in PC -9OR cells treated with osimertinib (Fig. 7b). Moreover, DAPT treatment sensitized EGFR-driven LUAD tumors to gefitinib (Fig. 7c–e) and osimertinib (Fig. 7g–i) without affecting the body weight of the mice (Figs. 7f, j). Remarkably, DAPT alone showed no significant antitumor effect in gefitinib- or osimertinib-resistant PC - 9 cells and PDX tumors (Figs. 7a, b, c–e, g–i). This is consistent with the above data that EGFR-TKI -induced STAT3 activation is required for NICD4-induced suppression of HES1 (Fig. 6d).

Because HES1 plays an important role in NICD4 regulation of LUAD sensitivity to EGFR-TKIs, we hypothesized that HES1 might also be an important target for sensitization of EGFR TKI treatment. Inhibition of HES1 signaling with JI051 increased the sensitivity of PC9- GR and PC - 9OR cells to gefitinib and osimertinib, respectively, after 72 hours of incubation in vitro (Fig. 7k, l). Gefitinib alone had no anti-tumor effect on PC - 9GR CDX tumors, but combined treatment with JI051 and gefitinib resulted in significant tumor inhibition compared with treatment with JI051 alone (Fig. 7m–o). A synergistic effect of treatment with JI051 and EGFR-TKI was observed in the suppression of PC −9OR CDX tumors (Fig. 7q–s), and there was no apparent effect on the body weight of the mice (Fig. 7p, t). We tested the common signaling pathways in the context of gefitinib resistance and found that the NOTCH4/HES1 pathway may mediate TKI resistance through effects on PI3K/Akt signaling. As shown in Supplementary Fig. 8, both DAPT and JI051 treatment significantly reduced the expression of genes in the PI3K/Akt pathway.

All in all, EGFR-TKI resistance in LUAD could be overcome by NOTCH4 or HES1 inhibitors. Therefore, they are expected to be potential targets for EGFR-TKI therapy.

## Discussion

EGFR TKIs are widely used to treat EGFR-related LUAD. However, treatment outcomes vary widely among patients, and only a small proportion of these patients continue to respond to EGFR-TKIs after 6 months of treatment. Therefore, more prognostic biomarkers are needed to predict the outcomes of EGFR-TKI treatment of EGFR-driven LUAD.

In this study, we found a NOTCH4^ΔL12_16^ mutation in approximately 18% of EGFR-driven LUAD patients and demonstrated that this mutation can be used to predict better outcomes with EGFR-TKI therapy. Functionally, the ΔL12_16 mutation in NOTCH4 suppressed translocation of this protein to the plasma membrane, thereby reducing NICD4 levels and downstream HES1 signaling. Both the introduction of exogenous NOTCH4^ΔL12_16^ and the reduction of NICD4 by siNOTCH4 increased the sensitivity of LUAD tumors or cells to EGFR-TKIs, and overexpression of HES1 reversed this enhancement. Of note, reduction of NICD4 did not suppress the growth of EGFR-driven LUAD tumors without treatment of EGFR-TKI, whereas it sensitized LUAD to EGFR-TKIs. Consequently, NICD4 did not directly transactivate HES1, although it could bind to its promoter. EGFR-TKI treatment can induce the activation of STAT3, which can inhibit the growth of EGFR-driven LUAD tumors by serving as a HES1 suppressor. The binding sites of p-STAT3 in the HES1 promoter were close to those of NICD4. Moreover, the depletion of NICD4 may enhance the binding of p-STAT3 to the HES1 promoter and suppress HES1 expression after EGFR-TKI treatment. Therefore, depletion of NICD4 sensitizes LUAD tumors to EGFR-TKIs by supporting the function of EGFR-TKI -induced p-STAT3 in suppressing HES1 signaling.

As shown in previous studies, the NOTCH signaling pathway plays an important role in the progression of LUAD^26–28^. The receptors NOTCH1, NOTCH2, and NOTCH3 play key roles in regulating the development of LUAD, whereas the functional role of NOTCH4 remains unclear^29–32^. We analyzed activated levels in tumor cells from EGFR-driven LUAD and found that only the activated form of NOTCH4 (NICD4) was greatly increased in tumor samples resistant to EGFR TKIs compared with those sensitive to EGFR TKIs. While NOTCH4^ΔL12_16^ was significantly decreased, mutational changes in other NOTCH proteins, including NOTCH1-3, were not detected during the progression of EGFR-TKI resistance in LUAD PDX tumors. These data would provide new insights into the role of the NOTCH pathway in regulating the sensitivity of LUAD tumors to EGFR TKIs.

To investigate whether targeting the NICD4/HES1 pathway can overcome the resistance of LUAD tumors to EGFR-TKIs, we used DAPT and JI051 to inhibit the production of NICD4 and the function of HES1, respectively, and to evaluate their roles in enhancing EGFR-TKI sensitivity in EGFR-driven LUAD tumors. Both DAPT and JI051 were able to resensitize EGFR- TKI-resistant LUAD tumors. These data suggest that DAPT and JI051 could be used to overcome drug resistance. Specifically, gefitinib treatment may induce an EGFR T790M mutation that causes drug resistance in LUAD tumors, and these tumors could be treated with osimertinib. However, only some gefitinib-resistant LUAD tumors carried the EGFR T790M mutation. For patients with gefitinib-resistant LUAD without EGFR T790M, treatment targeting NICD4/HES1 signaling may resensitize them to gefitinib. Therefore, our study provides new avenues for the treatment of patients with gefitinib-resistant LUAD. Moreover, the strategies of reducing NICD4 or suppressing HES1 may overcome resistance to osimertinib in LUAD tumors with EGFR T790M mutation, as siNOTCH4 can increase sensitivity to osimertinib in PC - 9OR cells carrying the T790M mutation of EGFR. Since p-STAT3 is required for NICD4 to suppress HES1 signaling and thus increase sensitivity to EGFR-TKIs in LUAD, the use of STAT3 activators to overcome EGFR-TKI resistance is worth exploring in the future. In addition, STAT3 inhibitors currently being tested in clinical trials^33,34^ should be used with caution, at least in tumors in which the NOTCH4 pathway and thus HES1 play a protumorigenic role, as in lung adenocarcinoma.

The doses of gefitinib and osimertinib administered in our in vivo experiments were within the clinically relevant range, as shown by previous studies^35–38^, but we are aware that the effects of these drugs may vary depending on patient characteristics and disease stage, and this may be one of the limitations of our study. In addition, the use of high concentrations of gefitinib in some of our in vitro experiments could be another limitation of our study. High concentrations of gefitinib may not accurately reflect the clinical situation, and further studies are needed to confirm our results in a more clinically relevant context.

Our findings may suggest new avenues for outcome prediction of EGFR-TKI therapy in LUAD and highlight a potential approach to sensitize EGFR-driven LUAD to TKIs.

## Methods

### Ethical statement

This study is approved by the ethics committee of Shenzhen People’s Hospital.

### Patients and tissue specimens

Fresh tumor samples were obtained from 10 patients diagnosed with EGFR-driven LUAD and treated at Shenzhen People’s Hospital from 2019 to 2022. Immediately after surgery, tumor samples were divided into three parts to create a PDX model, extract DNA/RNA, and perform pathological measurements. In addition, paraffin-embedded tumor samples from 44 EGFR-driven LUAD patients treated with EGFR-TKIs were used for DNA extraction and correlation analysis of NOTCH4^ΔL12_16^ mutation and efficacy of EGFR-TKI treatment. Written informed consent was obtained from each patient.

### Mice

NOD/ShiLtJGpt-Prkdcem26Cd52Il2rgem26Cd22/Gpt (NCG) and BALB/c nude female mice (4-week-old) were purchased from GemPharmatech Co., Ltd. (Nanjing, China) and housed in a specific pathogen-free (SPF) environment with a 12-hour light/dark cycle, temperature range of 20-26 °C, and a relative humidity range of 30–70%. The mice were housed in individually ventilated cages with food and water available ad libitum. Animal experiments were conducted in accordance with the laboratory guidelines for animal care and the protocols were approved by the Institutional Animal Care and Use Committee of Shenzhen People’s Hospital.

Humane endpoints for the mouse studies were preapproved in the study protocol by our institutional Animal Care and Use Committee (IACUC). The specific limits were determined based on factors such as the nature of the research and animal welfare. The maximum allowed tumor volume was set at 2000 mm³, and the maximum allowed weight loss was set at 15% of the pre-treatment body weight. These limits were established to ensure that the animals did not experience excessive pain or distress while allowing the experiments to reach a meaningful endpoint. If either of these criteria was exceeded, the corresponding humane endpoint measures, including euthanasia, were promptly executed.

### PDX model establishment

All fresh tumors were cut into pieces and set on a printer in a sterile environment to produce a “bio-ink”. A mixture of gelatin, sodium alginate, and bio-ink was printed layer by layer. A mixture of tumors and biomaterials was also injected into the subcutaneous flank of female NCG mice. Tumor size was measured every two days using a caliper. When the tumors reached a diameter of 1.0-1.5 cm, the mice were sacrificed and the tumors were implanted into new mice and transferred at least three times to ensure the stability of the model.

### Histological staining

Surgically resected tumors (SRT) from LUAD patients or PDX were fixed in formalin and embedded in paraffin. H&E staining was used to assess the pathology. For immunohistochemistry (IHC), 5μm-thick sections were treated with primary antibodies against human CK7 (Abcam, ab181598, 1:200), human CK5/6 (SIGMA, SAB5600242, 1:100), and human P63 (Abcam, ab124762, 1:100), human Syn (SIGMA, HPA018842, 1:200), human TTF-1 (SIGMA, SAB5500187, 1:100), and human Napsin A (Thermo Fisher Scientific, Z2294MS, 1:100). Cells were then incubated with secondary antibodies at room temperature and treated with the Vectastain ABC Kit (Vector Laboratories). The 3,3’-diaminobenzidine reaction was visualized by peroxidase activity. The IHC score was determined by three experienced pathologists at Shenzhen People’s Hospital.

### Whole exome sequencing (WES)

A total amount of 0.6 µg genomic DNA per sample was used as input material for the preparation of the DNA sample. Sequencing libraries were generated using the Agilent SureSelect Human All Exon kit (Agilent Technologies, CA, USA), following the manufacturer’s recommendations, and index codes were added to each sample. Briefly, fragmentation was performed using a hydrodynamic shearing system (Covaris, Massachusetts, USA) to generate 180–280 bp fragments. The remaining overhangs were converted into blunt ends via exonuclease/polymerase activity. After adenylation of the 3’ ends of the DNA fragments, the adapter oligonucleotides were ligated. DNA fragments with ligated adapter molecules at both ends were selectively enriched in PCR. After the PCR reaction, libraries hybridize with the liquid phase with a biotin-labeled probe and then use magnetic beads with streptomycin to capture the exons of the genes. Captured libraries were enriched in a PCR reaction to add index tags to prepare them for sequencing. Products were purified using the AMPure XP system (Beckman Coulter, Beverly, USA) and quantified using an Agilent high-sensitivity DNA assay on the Agilent Bioanalyzer 2100 system.

The clustering of the index-coded samples was performed on a cBot Cluster Generation System using a HiSeq PE Cluster Kit (Illumina) according to the manufacturer’s instructions. After cluster generation, the DNA libraries were sequenced on the Illumina HiSeq platform, and 150 bp paired-end reads were generated.

Valid sequencing data were mapped to the reference human genome (UCSC hg19) using the Burrows-Wheeler Aligner (BWA) software^39^ to obtain the original mapping results stored in BAM format. SAMtools^40^ and Picard (http://broadinstitute.github.io/picard/) were used to sort BAM files and perform duplicate marking, local realignment, and base quality recalibration to generate final BAM files for computation of sequence coverage and depth.

Samtools^40^, mpileup, and bcftools were used to perform variant calling and identify SNP and InDels. ANNOVAR^41^ was performed to annotate for VCF (variant call format) obtained in a previous study. dbSNP, 1000 Genome, and other related existing databases were used to characterize the detected variants. Somatic SNVs were detected using muTect^42^ and somatic InDel by Strelka^43^. Control-FREEC^44^ was used to detect somatic CNV.

### Cell culture and treatment

All cell lines were tested for mycoplasma contamination using a commercially available PCR-based assay and were confirmed to be negative for mycoplasma. The cells were maintained in culture according to standard procedures, and all experiments were performed using cells that were at low passage numbers (less than 20 passages from the original stock). Human lung cancer cells PC -9 were maintained in RPMI-1640 complete medium (BI, Israel) containing 10% fasting blood sugar (FBS) (5% CO2, 37 °C) and cultured according to the manufacturer’s protocol. Gefitinib and osimertinib (Selleck Chemicals, Houston, TX, USA) were dissolved in dimethyl sulfoxide (DMSO) to prepare stock solutions. Working concentrations were prepared by diluting the stock solution in RPMI-1640 medium with 10% fetal bovine serum (BI, Israel). The effect of gefitinib and osimertinib on cell proliferation was determined by counting viable cells with a colorimetric assay using Cell Counting Kit-8 (CCK8, Solarbio, China). Cells were seeded for 24 hours in 96-well plates with 3 × 10^3^ cells per well before treatment with gefitinib or osimertinib for 72 hours. The concentrations of gefitinib ranged from 0 to 64 μM (twofold dilution), and the concentrations of osimertinib (twofold dilution) ranged from 0 to 1 μM. And finally, the concentration of gefitinib was 10 μM and the concentration of osimertinib was 0.5 μM. The absorbance at 450 nm was measured using a spectrophotometer. Percentage of cytotoxicity was calculated using the following formula: % cytotoxicity = [1 - (absorbance of experimental well - absorbance of blank)/(absorbance of untreated control well - absorbance of blank)]× 100. The drug concentration required to inhibit cell growth by 50% (IC50) was determined from concentration-response curves generated using SPSS 19.0 software. Results are given as mean ± standard deviation (SD) of three independent experiments. Cell viability curves at different concentrations were generated using GraphPad Prism software (version 6.0).

### Treatments in mice

Approximately 5 × 10^6^ PC- 9GR or PC- 9OR cells were suspended in 0.15 mL PBS and injected subcutaneously into the back right flank region of 4-5 week-old, 18 g female BALB/c nude mice. Once the volume reached approximately 100 mm^3^ (5 days after tumor inoculation), the mice were randomly assigned to different treatment groups (6 mice in each group). PC-9GR CDX or PDX-GRs were treated with gefitinib (75 mg/kg/d, oral administration for 21 days), DAPT (100 mg/kg s.c.), JI051 (25 mg/kg/d, p.o. for 14 days), siNOTCH4, siSTAT3, or their combination. For PC-9OR, CDX or PDX-ORs, the treatment contained osimertinib (5 mg/kg/d, p.o. for 14 days), DAPT (100 mg/kg s.c.), JI051 (25 mg/kg/d, p.o. for 14 days), siNOTCH4, siSTAT3, or combinations. The treatment was administered twice daily. Body weight and tumor volume (V) were recorded every 2 days. V was calculated as 0.5 × length × width^2^. Finally, the mice were sacrificed and the tumors were isolated for measurement and weighing.

### Plasmid transfection and RNA interference

Plasmid transfection and RNA interference 293 T or PC -9GR/ OR cells were plated 1 day before transfection and were approximately 60-70% confluent at the time of transfection. The indicated amounts of plasmid DNA were added to 100 μl Opti- MEM (Gibco/ BRL) and incubated with Lipofectin reagent (6 μl/1 μg DNA; Invitrogen) for 15 minutes at room temperature and then added to the cells. Cells were incubated with the transfection mixture for 5 hours before normal medium was added. Cells were harvested 48 hours after transfection. Cell lysates were assayed for luciferase activity using a luciferase assay system (Promega). Luciferase activity was normalized to the protein content of the lysates, which was determined by a Bradford assay (Bio-Rad) using γ-globulin as a standard.

siRNAs against NOTCH-4 or β-catenin were designed and chemically synthesized (Sangon Biotech Co., Shanghai, China) to target different coding regions of this gene, as described previously^45,46^.

### Western blot analysis

Treated cells and tumors were lysed in RIPA buffer, and proteins were quantified by the BCA method. Samples were separated with 10% SDS-PAGE and transferred to polyvinylidene fluoride (PVDF) membranes. The membranes were blocked in 5% skim milk for 2 hours and incubated overnight with the diluted (1:1000) primary antibodies at 4°C: NOTCH4 full length (Abcam, ab166605, 1:500), NOTCH4 (NICD4) (CST, #2423), HES1 (CST, #11988), PI3K (CST, #4292), Akt (CST, #4691), phospho-Akt (Ser473) (CST, #4060), MAPK (CST, #9102), P21 (CST, #2947), HEY1 (CST, #5315), phospho-STAT3(Tyr705) (CST, #9145), STAT3 (CST, #9139), NF-κB (CST, #8242), JAK1 (CST, #3332) and GAPDH (CST, #5174). After washing three times with TBST, membranes were incubated with a secondary antibody: HRP-conjugated goat anti-rabbit antibody and goat anti-rabbit antibody IgG (1:5,000, Abcam Inc., Cambridge, CA, USA) for 1.5 h at room temperature. Membranes were washed with TBST and developed using SuperSignal™ West Femto Maximum Sensitivity Substrate (Thermo Fisher Scientific, Waltham, USA).

### ChIP

Tumor cells derived from PDX, 293 T, or PC -9GR/ OR cells were incubated in a culture plate with medium containing 0.4% (for histone modification analysis) or 1% (for AP−1 occupancy analysis) formaldehyde for 10 minutes at room temperature. The cross-linking reaction was terminated by incubation with 0.125 M glycine for 5 minutes. Cells were harvested by scraping, collected by centrifugation at 400 × *g* for 8 min, and washed in phosphate-buffered saline (PBS). Nuclei were isolated by incubation in cell lysis buffer (10 mM Tris, 10 mM NaCl, and 0.2% NP −40 [pH 8.0]) on ice for 10 min, followed by centrifugation at 500 × *g* for 5 min. Nuclei were lysed in nuclear lysis buffer (50 mM Tris, 10 mM EDTA, and 1% sodium dodecyl sulfate [SDS] [pH 8.0]) for 10 min on ice. The lysate was diluted with IP dilution buffer (20 mM Tris, 150 mM NaCl, 2 mM EDTA, 0.01% SDS, and 1% Triton X-100 [pH 8.0]) and sonicated with eight 30-s pulses at 50–60% of maximum power using a HeatWave Systems W185F Sonicator (Ultrasonics, Inc., Plainview, N.Y.) equipped with a microtip. The sonicated chromatin fragments had an average size of approximately 300–400 bp. Soluble chromatin was pre-purified by the addition of 50 μl pre-immune serum followed by 100 μl protein A-Sepharose. Pre-purified chromatin (180 μl) was removed (input) and used for subsequent PCR analysis. The remaining chromatin was aliquoted and incubated with the indicated antibodies in a final volume of 900 μl for 3 hours at 4 °C. Immune complexes was collected by incubation with 30 μl protein A-Sepharose for 2 hours at 4 °C. Protein A-Sepharose pellets were incubated twice with 500 μl aliquots of IP wash buffer 1 (20 mM Tris, 50 mM NaCl, 2 mM EDTA, 0.1% SDS, 1% Triton X-100 [pH 8.0]), once with IP wash buffer 2 (10 mM Tris, 0.25 mM LiCl, 1 mM EDTA, 1% NP −40, 1% deoxycholate [pH 8.0]), and twice with TE (10 mM Tris, 1 mM EDTA [pH 8.0]). Immune complexes were eluted twice with 150 μl IP elution buffer (0.1 mM NaHCO3, 1% SDS). RNase A (0.03 mg/ml) and NaCl (0.3 m/l) were added, and crosslinks were reversed by incubation for 4-5 hours at 65 °C. The DNA was then digested with proteinase K (0.24 mg/ml) for at least 2 hours at 45 °C and purified by two extractions with phenol-chloroform followed by ethanol precipitation. The purified DNA was resuspended in 30 μl of water. Aliquots (1 μl) were analyzed by quantitative real-time PCR using the indicated primer pairs. The amount of product was determined relative to a standard curve generated from the titration of the input chromatin. Anti-NOTCH4 antibody was purchased from CST (catalog no. 2423) and anti-phospho-STAT3 was purchased from Cell Signaling Technology (catalog no. 9145). Immunoprecipitated DNA was analyzed by PCR using the primers PromHES1 forward (GAAGGCAATTTTTCCTTTC) and PromHES1 reverse (AAGTTCCCGCTCAGACTTTAC).

### Statistical analysis

Data were analyzed with the R package (version 3.3.0) or Prism 6.0 (Graph Pad Software Inc, La Jolla, CA, USA). Fisher’s exact test was used to compare the proportions between the 2 groups. Correlations of mutation prevalence were examined using Pearson’s method. A two-sided *P* < 0.05 was considered statistically significant.

### Reporting summary

Further information on research design is available in the Nature Portfolio Reporting Summary linked to this article.

## Supplementary information

Supplementary Information

Reporting Summary

## Data Availability

Raw WES data generated in this study have been deposited in Genome Sequence Archive for human online database under the accession number HRA002758. This raw WES data is currently under controlled access for 1 year due to patient privacy considerations and could be downloaded upon request through GSA website for academic purposes only. The human reference genome (UCSC hg19) publicly available data used in this study are available in the UCSC database (ftp hgdownload.soe.ucsc.edu). The remaining data are available within the Article, Supplementary Information or Source Data file. Source data are provided with this paper.

## Source data

Source Data
