# Supplementary material for: NOTCH4ΔL12_16 sensitizes lung adenocarcinomas to EGFR-TKIs through transcriptional down-regulation of HES1
Source: Nat Commun. 2023 Jun 2;14:3183. doi: 10.1038/s41467-023-38833-7 (PMC10238419; doi:10.1038/s41467-023-38833-7)
Supplement: Supplementary file 1 — Supplementary Information [file 41467_2023_38833_MOESM1_ESM.pdf]

Supplementary Information

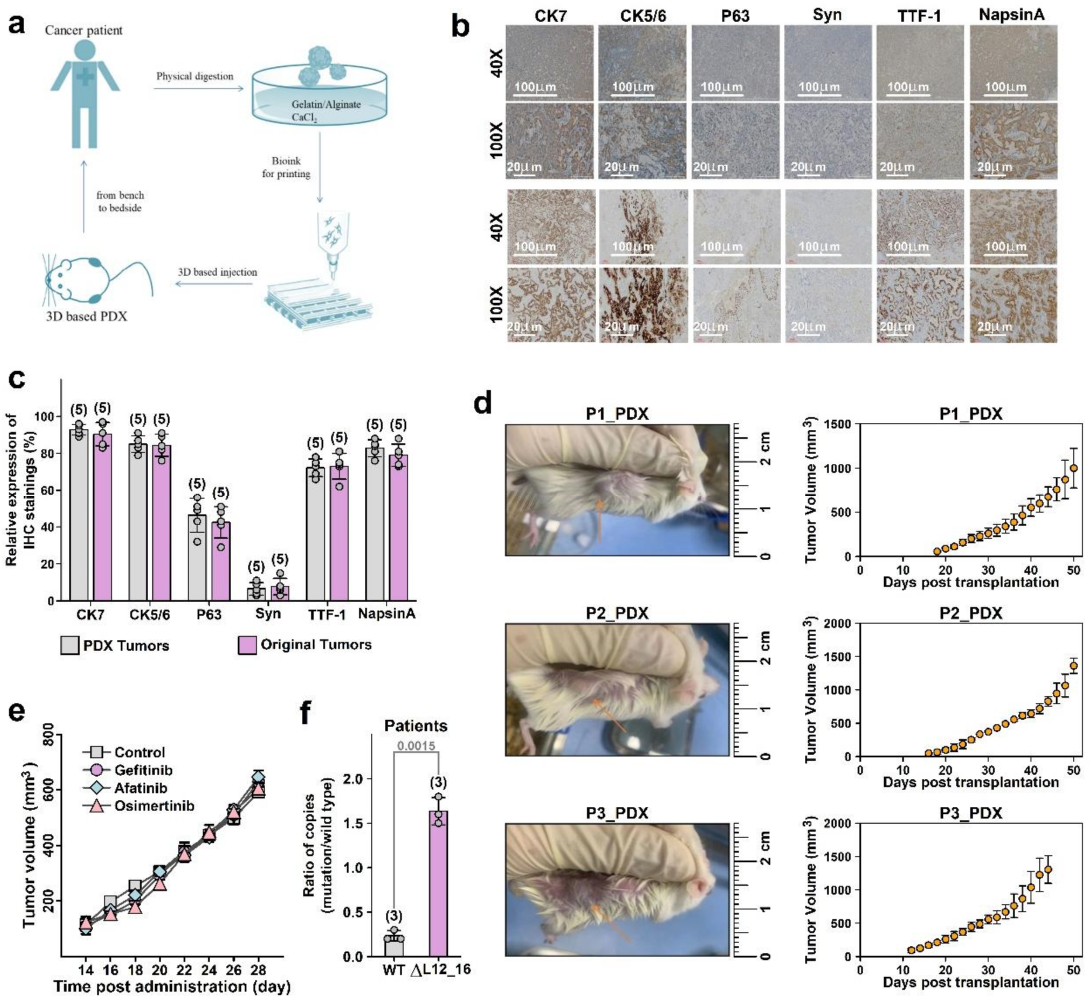

Supplementary Fig. 1. 3D based PDX construction and identification

(**a**) The flow chart of 3D based PDX models construction.

(**b-d**) The IHC analysis of PDX tumors and original tumors and the growth curve of P1 - P3 PDX models. (n = 5 Tumors for Figure S1C; n = 3 PDXs for Figure S1D)

(**e**) The tumor growth of EGFR - TKIs resistant PDX models with EGFR - TKIs treatments. (n = 3 PDXs)

(**f**) The ratio of mutation copies in LUAD patients compared with wild type. (n = 3 Patients)

Data are presented as mean ± SD. An unpaired two.sided student t.test was performed in Supplementary Fig. 1f. Source data are provided as a Source Data file.

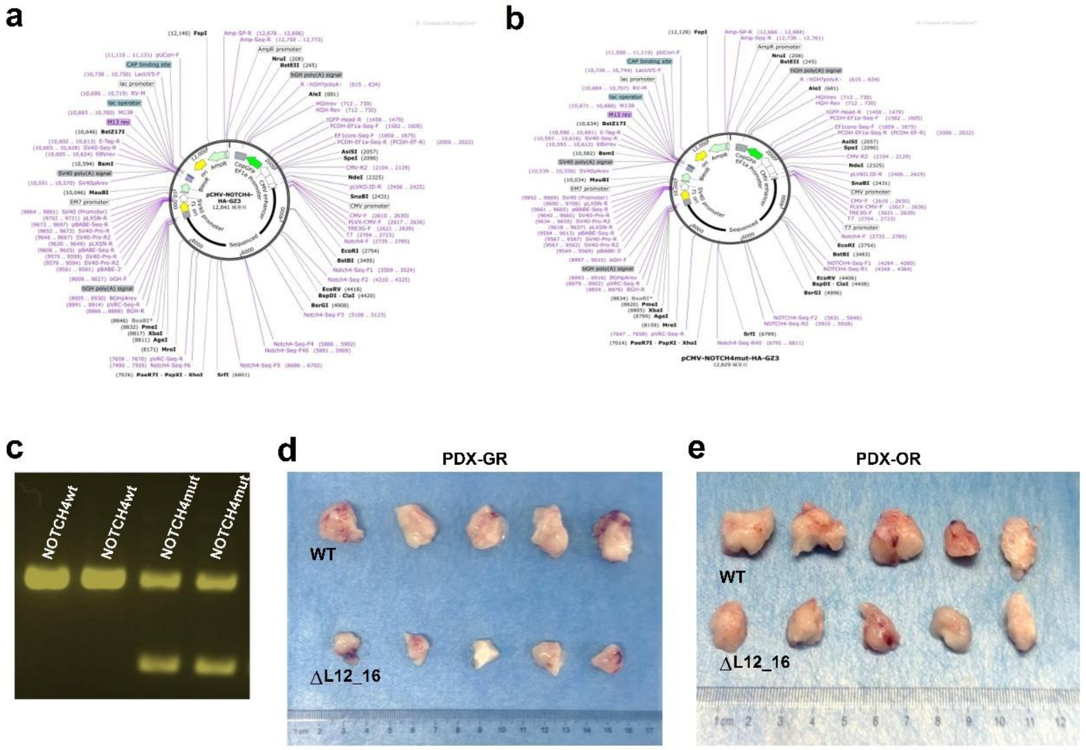

Supplementary Fig. 2. NOTCH4 plasmids atlas and identification.

(**a and b**) The atlas of wild type and mutation of NOTCH4 plasmids.

(**c**) The PCR verification of NOTCH4 mutation plasmids transfected.

(**d and e**) The tumor pictures with NOTCH4 mutation plasmids transfection into PDX-GR / PDX-OR models.

3 biologically independent experiments were performed for Supplementary Fig. 2c. Source data are provided as a Source Data file.

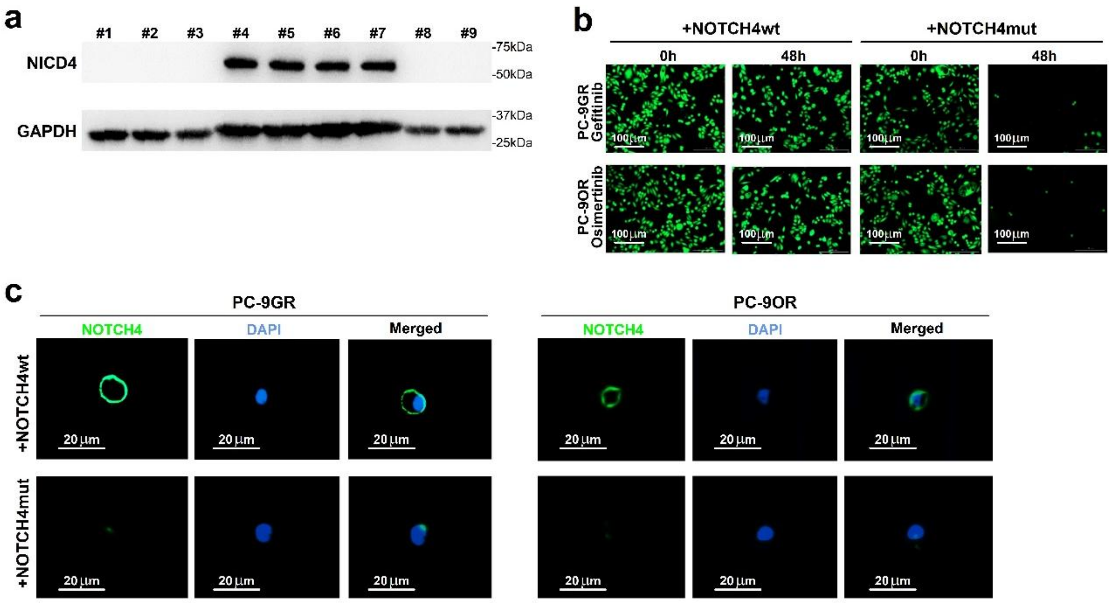

Supplementary Fig. 3. NICD4 expression in LUAD patients and ΔL12_16 transfected cells

(**a**) The NICD4 protein expression in LUAD patients. 1, 2, 3, 8 and 9 were showed as NICD4 low expression and others were high expression.

(**b and c**) The fluorescence results of PC-9GR / PC-9OR with geftinib or osimertinib treatment after NOTCH4 plasmids transfection.

Source data are provided as a Source Data file.

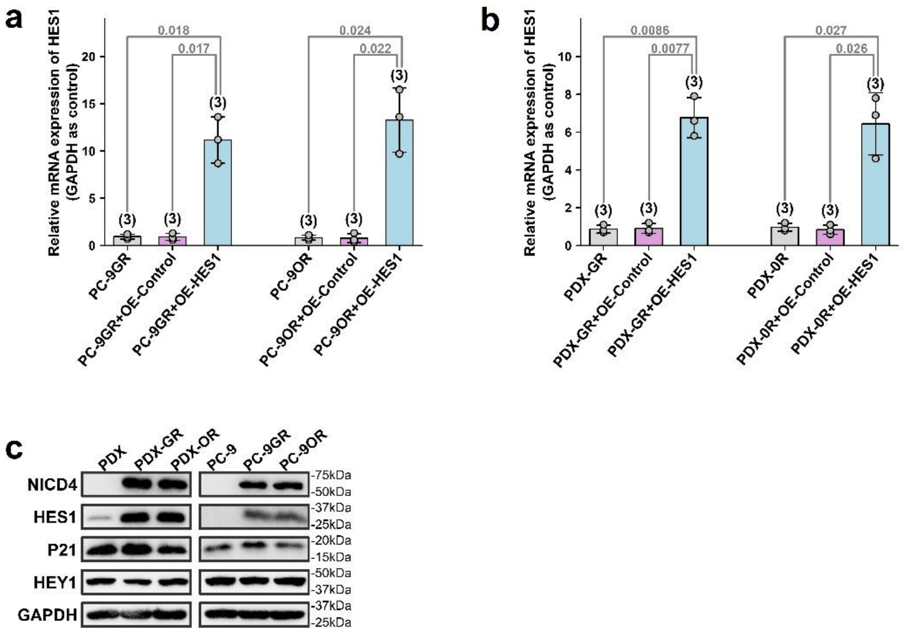

Supplementary Fig. 4. *HES1* over expression in EGFR-TKIs resistant cells

(**a and b**) The relative mRNA of HES1 expression. (n = 3 biologically independent experiments)

(**c**) The western blot analysis of PDX models and PC-9 / PC-9GR /PC-9OR cells.

Data are presented as mean ± SD. An unpaired two.sided student t.test was performed in Figure S4A and S4B. Source data are provided as a Source Data file.

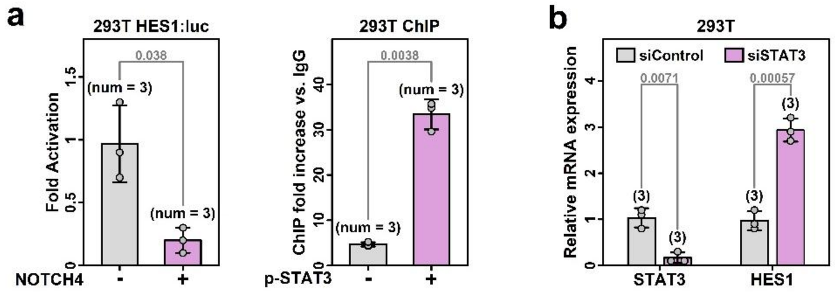

Supplementary Fig. 5. p-STAT3 binds to HES1 and Human *HES1* promoter sequences

(**a**) Luciferase analysis of NOTCH4 to HES1 with p-STAT3 treatment and ChIP analysis of p-STAT3 binding to the *HES1* promoter in 293T cells. (n = 3 biologically independent experiments)

(**b**) The relative mRNA expression of NOTCH4 and STAT3 with siSTAT3 treatment. (n = 3 biologically independent experiments)

Data are presented as mean ± SD. An unpaired two.sided student t.test was performed in Supplementary Fig. 5a and 5b. Source data are provided as a Source Data file.

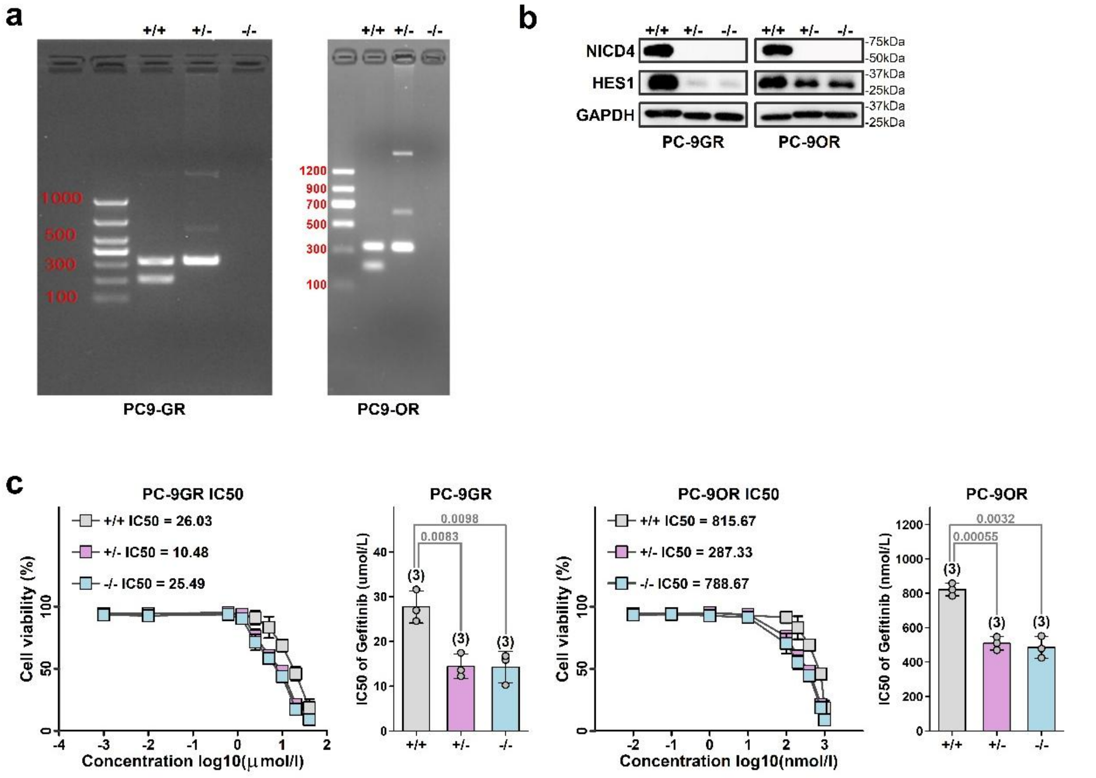

Supplementary Fig. 6.

(**a**) Verification of gene knockout using PCR;

(**b**) IC50 in DeltaL12-16 NOTCH4+/+, DeltaL12-16 NOTCH4+/- and DeltaL12-16 NOTCH4-/-;

(**c**) Relative protein expression in DeltaL12-16 NOTCH4+/+, DeltaL12-16 NOTCH4+/- and DeltaL12-16 NOTCH4-/-. (n = 3 biologically independent experiments)

Data are presented as mean ± SD. An unpaired two.sided student t.test was performed in Supplementary Fig. 6c. Source data are provided as a Source Data file.

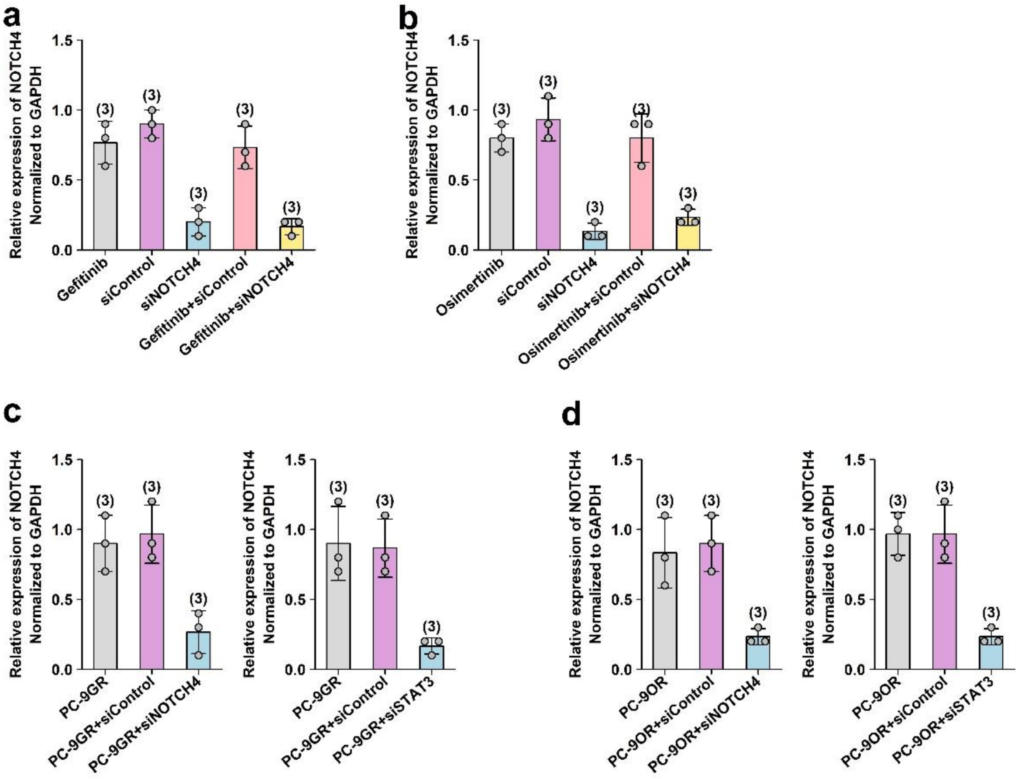

Supplementary Fig. 7. Verifications of gene knowndown using Realtime PCR

(**a**) Verification of NOTCH4 knockdown (corresponding to Main Figure 4F); (n = 3 biologically independent experiments)

(**b**) Verification of NOTCH4 knockdown (corresponding to Main Figure 4I); (n = 3 biologically independent experiments)

(**c**) Verification of NOTCH4 and STAT3 knockdown (corresponding to Main Figure 6F); (n = 3 biologically independent experiments)

(**d**) Verification of NOTCH4 and STAT3 knockdown (corresponding to Main Figure 6G); (n = 3 biologically independent experiments)

Data are presented as mean ± SD. Source data are provided as a Source Data file.

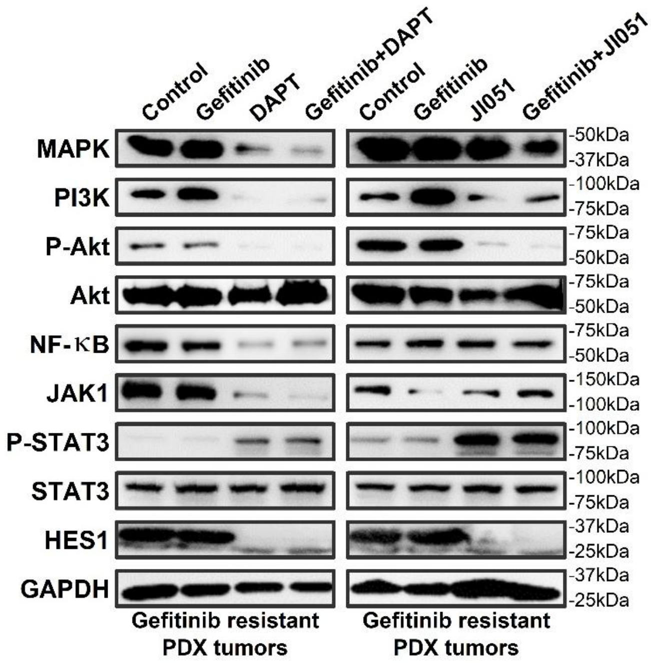

Supplementary Fig. 8. Relative protein expression levels of EGFR-TKI resistance related genes after inhibition of NOTCH4 or HES1

Source data are provided as a Source Data file.

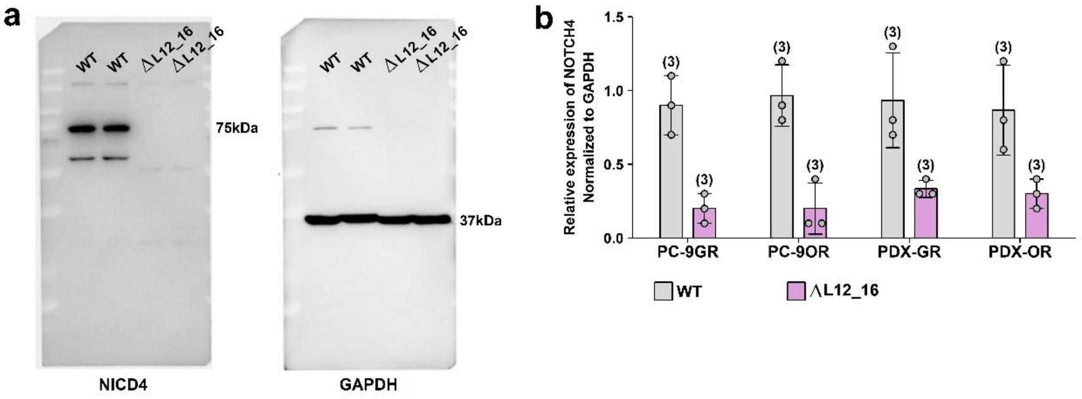

Supplementary Fig. 9. Expression levels of wild type and mutant NOTCH4 in different cell lines.

(**a**) Verification of NOTCH4 knockdown by westernblot (corresponding to Main Figure 2);

(**b**) Verification of NOTCH4 knockdown by real-time PCR (corresponding to Main Figure 2) (n = 3 biologically independent experiments)

Data are presented as mean ± SD. Source data are provided as a Source Data file.

Supplementary Table 1. The gene mutation frequency in patients' tumor, PDX models and PC-0/PC-9GR/PC-9OR cells

	Patients		PDX-GR		PDX-OR		PC-9		PC-9GR		PC-9OR	
	SYN	MIS	SYN	MIS	SYN	MIS	SYN	MIS	SYN	MIS	SYN	MIS
NOTCH4	85	84	88	3	82	2	92	82	85	3	87	2
NOTCH3	91	82	85	87	83	86	91	88	85	87	83	86
NOTCH2	88	83	82	88	81	85	93	83	82	88	81	85
NOTCH1	87	82	86	89	85	86	94	82	86	89	85	86
ALK	88	82	86	85	88	87	92	82	86	87	88	83
BRAF	92	6	88	6	84	3	91	6	88	4	79	3
EGFR	93	88	85	83	82	89	96	88	85	86	82	89
ERBB2	83	87	84	82	82	84	85	87	84	82	82	84
KRAS	86	8	83	9	81	6	89	8	79	6	81	6
MET	82	6	91	5	93	6	88	6	88	5	93	4
RET	81	85	86	84	84	82	83	85	86	87	84	82
ROS1	86	82	85	85	85	81	86	82	85	85	85	85
MAP2K1	83	88	82	86	87	83	85	84	82	86	87	83
PIK3CA	88	6	81	8	86	7	88	6	81	8	86	5
NTRK1	89	4	88	6	82	5	89	4	86	6	82	5
OTHERS	93	95	91	94	92	97	93	91	93	95	92	91

Supplementary Info 1. Human *HES1* promoter sequences. The consensus binding sites for RBPJ and STAT3 are highlighted in red and yellow, respectively. Red letters indicate where the coding sequences (CDS) of both genes start.

acgccggccggctgatgtcaaactgcagctcggctggtgtagctcttaaa
gggcccgcgggcgccgggggccgaggccgcccgcggggcgaggagggaga
ggctgcttccttgcccgccacctcaacgcgccgcagaacctaaagcctac
ggatgaaaagggaaagggtggtgaaggagggcgcgagtccctgagtcatt
gccctcggagcccccccttgttcatttttcatttgaaataccgtttcctt
ccacccagttggaaattattctgattgtttcctggggaagcccggggtct
aaggccccaaatccaaacgaggaatttctgaaagacggggtgggggtggg
attcaagaactaccttgctccgaaaaacctgcatttgtgaggtagaaggc
aatttttcctttttctgcatggaaacaggaaaattttttggccctttttc
ctttaccatctactttcaccctcctgaatgtaaagtctgagcgggaactt
tagatgtgtcggtaactcacattcttacacccgtcccccccctccccccgc
cccctttttaaccactgctgtttttttctttattgtttatacctttaaaaa
aaatatgtttcaaatgaacttactacagtcaaagcagctctgttacatat
gagagagggcataaagagcaaagaccctggctccaaaagaaatagacaag
atcaagaccaaagcggaaagaaaaaaaaaatctctaaaccaaagcccaga
gggagagtagcaaagggttaaaatccttttgattgacgttgtagcctccg
gtgccctgggctcaggcgcgcgccattggccgccagaccttgtgcctggc
ggccaatgggggggcgcggtccacgagcggtgccgcgtgtctcctcctcc
cattggctgaaagttactgtgggaaagaaagtttgggaagtttcacacga
gccgttcgcgtgcagtcccagatatatatagaggccgccagggcctaggg
ATCACACAGGATCCGGAGCTGGTGCTGATAACAGCGGAATCCCCCGTCTA
CCTCTCTCCTTGGTCCTGGAACAGCGCTACTGATCACCAAGTAGCCACAA
AATATAATAAACCCTCAGCACTTGCTCAGTAGTTTTGTGAAAGTCTCAAG
TAAAAGAGACACAAACAAAAAATTCTTTTTCGTGAAGAACTCCAAAAATA
AAATTCTCTAGAGATAAAAAAAAAAAAAAAAAGGAAAATGCCAGCTGATAT

STAT3
PBPJ
