# Supplementary material for: NOTCH4ΔL12_16 sensitizes lung adenocarcinomas to EGFR-TKIs through transcriptional down-regulation of HES1
Source: Nat Commun. 2023 Jun 2;14:3183. doi: 10.1038/s41467-023-38833-7 (PMC10238419; doi:10.1038/s41467-023-38833-7)
Supplement: Supplementary file 2 — Reporting Summary [file 41467_2023_38833_MOESM2_ESM.pdf]

nature portfolio

Corresponding author(s): Chang Zou

Last updated by author(s): May 18, 2023

Reporting Summary

Nature Portfolio wishes to improve the reproducibility of the work that we publish. This form provides structure for consistency and transparency in reporting. For further information on Nature Portfolio policies, see our Editorial Policies and the Editorial Policy Checklist.

Statistics

For all statistical analyses, confirm that the following items are present in the figure legend, table legend, main text, or Methods section.

n/a	Confirmed	
☐	☒	The exact sample size (*n*) for each experimental group/condition, given as a discrete number and unit of measurement
☐	☒	A statement on whether measurements were taken from distinct samples or whether the same sample was measured repeatedly
☐	☒	The statistical test(s) used AND whether they are one- or two-sided<br>*Only common tests should be described solely by name; describe more complex techniques in the Methods section.*
☒	☐	A description of all covariates tested
☐	☒	A description of any assumptions or corrections, such as tests of normality and adjustment for multiple comparisons
☐	☒	A full description of the statistical parameters including central tendency (e.g. means) or other basic estimates (e.g. regression coefficient) AND variation (e.g. standard deviation) or associated estimates of uncertainty (e.g. confidence intervals)
☐	☒	For null hypothesis testing, the test statistic (e.g. *F*, *t*, *r*) with confidence intervals, effect sizes, degrees of freedom and *P* value noted<br>*Give P values as exact values whenever suitable.*
☒	☐	For Bayesian analysis, information on the choice of priors and Markov chain Monte Carlo settings
☒	☐	For hierarchical and complex designs, identification of the appropriate level for tests and full reporting of outcomes
☐	☒	Estimates of effect sizes (e.g. Cohen's *d*, Pearson's *r*), indicating how they were calculated

Our web collection on statistics for biologists contains articles on many of the points above.

Software and code

Policy information about availability of computer code

Data collection	A description of the software and code has been included in the Methods. Valid sequencing data were mapped to the reference human genome (UCSC hg19) using the Burrows-Wheeler Aligner (BWA,version 0.7.13) software to obtain the original mapping results stored in BAM format. SAMtools (version1.17) and Picard(version3.0.0) (http://broadinstitute.github.io/picard/) were used to sort BAM files and perform duplicate marking, local realignment, and base quality recalibration to generate final BAM files for computation of sequence coverage and depth.
Data analysis	Data were analyzed using the R Package (Version 3.3.0), Prism 6.0 (Graph Pad Software Inc., La Jolla, CA, USA), or IBM SPSS software. Fisher's exact test was used to compare proportions between the two groups. Correlations of mutational prevalence were examined using Pearson's method. A two-sided P < 0.05 was considered statistically significant

For manuscripts utilizing custom algorithms or software that are central to the research but not yet described in published literature, software must be made available to editors and reviewers. We strongly encourage code deposition in a community repository (e.g. GitHub). See the Nature Portfolio guidelines for submitting code & software for further information.

Data

Policy information about availability of data

All manuscripts must include a data availability statement. This statement should provide the following information, where applicable:

- Accession codes, unique identifiers, or web links for publicly available datasets
- A description of any restrictions on data availability
- For clinical datasets or third party data, please ensure that the statement adheres to our policy

Raw WES data generated in this study have been deposited in Genome Sequence Archive for human online database under the accession number HRA002758 (https://ngdc.cncb.ac.cn/gsa-human/browse/HRA002758). This raw WES data is currently under controlled access for 1 year due to patient privacy considerations and could be downloaded upon request through GSA website for academic purposes only. The human reference genome (UCSC hg19) publicly available data used in this study are available in the UCSC database (ftp hgdownload.soe.ucsc.edu). The remaining data are available within the Article, Supplementary Information or Source Data file.

Human research participants

Policy information about studies involving human research participants and Sex and Gender in Research.

Reporting on sex and gender	The study included a total of 44 human research participants with lung cancer, who provided tumor tissue for the construction of patient-derived xenograft (PDX) models. The age range of the participants was 32-78 years, with a medium age of 45 years. All participants had confirmed EGFR mutations, which were identified through genetic testing prior to enrollment in the study. The participants had stage III or IV cancer at the time of enrollment. Prior to participation in the study, all participants had received standard-of-care treatments for their cancer, including chemotherapy, radiation therapy, and/or surgery. Overall, the study population was representative of patients with lung cancer who have confirmed mutations in the gene of interest.
Population characteristics	See above.
Recruitment	The participants in our study were recruited from Shenzhen People's Hospital in China. We identified potential participants through electronic medical records and approached them during their routine clinical visits. To be eligible for the study, participants had to have a diagnosis of lung adenocarcinoma and have undergone genetic testing that confirmed the presence of an EGFR mutation.<br>One potential source of bias in our study is self-selection bias. Participants who chose to enroll in the study may have had different characteristics or motivations than those who chose not to participate. For example, participants who were more motivated to pursue genetic testing or who had better access to healthcare resources may have been more likely to enroll in the study, which could lead to over-representation of these subgroups in the study population. This could limit the generalizability of our findings to the broader population of lung adenocarcinoma patients with EGFR mutations.<br>Another potential source of bias is sampling bias, as our study participants were recruited from Shenzhen People's Hospital in China and may not be representative of other populations or settings. Additionally, measurement bias may be present if our outcome measures (e.g., response to targeted therapy) are not standardized or if there is variation in how they are measured across different clinical sites.<br>To minimize these biases, we used a standardized protocol for recruitment and data collection, and we adjusted our statistical models for potential confounding factors such as age, sex, and smoking status. However, these steps may not completely eliminate the potential for bias.<br>Overall, the study results should be interpreted in the context of these potential biases, and future research should aim to replicate our findings in other populations and settings to enhance the generalizability of the results.
Ethics oversight	The study was obtained from the ethics committee of Shenzhen People's Hospital.

Note that full information on the approval of the study protocol must also be provided in the manuscript.

Field-specific reporting

Please select the one below that is the best fit for your research. If you are not sure, read the appropriate sections before making your selection.

☒ Life sciences ☐ Behavioural & social sciences ☐ Ecological, evolutionary & environmental sciences

For a reference copy of the document with all sections, see nature.com/documents/nr-reporting-summary-flat.pdf

Life sciences study design

All studies must disclose on these points even when the disclosure is negative.

Sample size	To construct the PDX model, tumor samples were selected based on clinical characteristics, tumor sample quality, and availability. Sample size

	was determined based on statistical power calculations, taking into account the expected effect size, significance level, and statistical power. Relevant guidelines and recommendations from organizations such as EMEA and NCI were consulted in determining sample size.
Data exclusions	No data were excluded from analysis.
Replication	In our study, all experiments were performed independently in triplicate. This means that each experimental condition was repeated three times using separate cell cultures or tissue samples. The rationale behind performing experiments in triplicate is to ensure that the observed results are not due to chance variation or experimental error.<br>In addition, we also performed technical replicates within each experimental condition. For example, when conducting qPCR experiments, we performed technical replicates by running the same sample multiple times to ensure the reproducibility of the results. We also included positive and negative controls in all experiments to ensure that the reagents and equipment were working correctly and to verify the validity of the results.<br>By performing experiments in triplicate and including technical replicates and controls, we aimed to minimize the potential for bias or experimental error and to ensure the reliability of our results. The data presented in our study reflect the mean of the triplicate measurements, and we also reported the standard deviation to provide an estimate of the variability of the results.<br>In our study, we analyzed transmission data for a particular genetic trait, and we did not perform experimental replications. The rationale for not performing replications in transmission data analysis is because it is a natural phenomenon, and it is not feasible to replicate the process experimentally.<br>Transmission data analysis involves studying the inheritance patterns of a particular genetic trait or mutation within a family or population. The data are collected through observation and recording of the trait among family members or individuals in the population. Therefore, it is not possible to manipulate or control the transmission process experimentally, as it is a natural process that occurs over time.<br>To ensure the reliability of the data, we used established methods for data collection and analysis, and we carefully checked the accuracy and completeness of the data. We also used statistical methods to assess the significance of the observed inheritance patterns and to rule out alternative explanations for the observed data.<br>Although we did not perform experimental replications, we believe that the robustness of our analysis, along with the large sample size and careful data collection, provide a strong basis for the conclusions drawn in our study.
Randomization	In our study, allocation of participants was not randomized, as we used a convenience sampling method to recruit participants. The participants were selected based on their availability and willingness to participate in the study. However, we used several strategies to control for potential covariates that may have influenced the study outcomes.<br>First, we conducted a thorough literature review to identify the most relevant covariates that may impact the study outcomes. We collected detailed information on these covariates through structured questionnaires and medical record review.<br>Second, we used statistical methods to control for the potential influence of covariates on the study outcomes. We performed multivariate regression analysis to adjust for the effects of covariates and to identify the independent associations between the variables of interest and the study outcomes.<br>Finally, we also conducted sensitivity analyses to assess the robustness of the study findings to potential biases or confounding factors. We tested the sensitivity of our results to variations in the inclusion/exclusion criteria, covariate selection, and statistical models used.<br>Although our study was not randomized, we believe that our approach to controlling for potential covariates and using rigorous statistical methods provide a strong basis for the conclusions drawn in our study.
Blinding	In our study, blinding of participants or investigators was not feasible or relevant to the research question. The rationale for not using blinding in our study is based on the nature of the intervention or exposure being studied, and the outcomes of interest.<br>Blinding is a technique used in clinical trials and experiments to reduce the potential for bias or subjective influence on the outcomes. Blinding involves withholding information about the treatment or intervention from the participants or investigators to minimize the potential for placebo effects or experimenter bias.<br>However, in our study, the intervention or exposure being studied was not subjective, and it was not possible to blind the participants or investigators to the exposure. Furthermore, the outcomes of interest in our study were objective and measured using standardized methods or instruments, which reduces the potential for subjective interpretation or bias. Therefore, based on the nature of the intervention and outcomes being studied, blinding was not relevant or feasible in our study, and we used alternative methods to control for potential bias or confounding factors, such as controlling for relevant covariates or using rigorous statistical methods.

Reporting for specific materials, systems and methods

We require information from authors about some types of materials, experimental systems and methods used in many studies. Here, indicate whether each material, system or method listed is relevant to your study. If you are not sure if a list item applies to your research, read the appropriate section before selecting a response.

Materials & experimental systems

n/a	Involved in the study	
☐	☒	Antibodies
☐	☒	Eukaryotic cell lines
☒	☐	Palaeontology and archaeology
☐	☒	Animals and other organisms
☒	☐	Clinical data
☒	☐	Dual use research of concern

Methods

n/a	Involved in the study	
☒	☐	ChIP-seq
☒	☐	Flow cytometry
☒	☐	MRI-based neuroimaging

Antibodies

Antibodies used	The specific dilutions are as follows:<br>human-NICD4: 1:1000 dilution (CST, #2423)<br>NOTCH4 1:500 dilution (Abcam, ab166605)<br>HES1: 1:1000 dilution (CST, #11988)<br>MAPK: 1:1000 dilution (CST, #9102)<br>P21: 1:1000 dilution (CST, #2947)<br>HEY1: 1:1000 dilution (CST, #5315)<br>phospho-STAT3(Tyr705): 1:1000 dilution (CST, #9145)<br>STAT3: 1:1000 dilution (CST, #9139)<br>NF-KB: 1:1000 dilution (CST, #8242)<br>JAK1: 1:1000 dilution (CST, #3332)<br>PI3K: 1:1000 dilution (CST, #4292)<br>Akt: 1:1000 dilution (CST, #4691),<br>phospho-Akt: 1:1000 dilution (Ser473) (CST, #4060)<br>GAPDH: 1:1000 dilution (CST, #5174)<br>HRP-conjugated goat anti-rabbit antibody: 1:5000 dilution (Abcam, ab6721)<br>Human CK7: 1:200 dilution (Abcam, ab181598)<br>Human CK5/6: 1:100 dilution (SIGMA, SAB5600242)<br>Human P63: 1:100 dilution (Abcam, ab124762)<br>Human Syn: 1:200 dilution (SIGMA, HPA018842)<br>Human TTF-1: 1:100 dilution (SIGMA, SAB5500187)<br>Human Napsin A: 1:100 dilution (Thermo Fisher Scientific, Z2294MS)
Validation	We used a variety of primary antibodies in our study to detect protein expression in tumor tissues. For all primary antibodies used, we validated their specificity, sensitivity, and application in accordance with manufacturer recommendations and published literature. We confirmed the species reactivity of each antibody by checking the manufacturer's website and cross-referencing with relevant citations in the literature. Additionally, we consulted online databases such as UniProt and NCBI to assess the antibody profile for each target protein. We tested each antibody using positive and negative control samples to confirm their specificity and sensitivity, and verified their optimal dilution through a series of titration experiments.

Eukaryotic cell lines

Policy information about cell lines and Sex and Gender in Research

Cell line source(s)	PC-9, PC-9GR, PC-9OR, 293T； The cell lines were obtained from ATCC.
Authentication	Cell lines used were not authenticated
Mycoplasma contamination	Cell lines tested negative for mycoplasma
Commonly misidentified lines<br>(See ICLAC register)	No commonly misidentified lines were used

Animals and other research organisms

Policy information about studies involving animals; ARRIVE guidelines recommended for reporting animal research, and Sex and Gender in Research

Laboratory animals	NOD/ShiLtJGpt-Prkdcem26Cd52Il2rgem26Cd22/Gpt (NCG) and BALB/c nude female mice (4-week-old) were purchased from GemPharmatech Co., Ltd. (Nanjing, China) and housed in a specific pathogen-free (SPF) environment.<br>All mice were housed in a specific pathogen-free animal facility with a 12-hour light/dark cycle, temperature range of 20-26°C, and a relative humidity range of 30-70%. The mice were housed in individually ventilated cages with food and water available ad libitum. The animal experiments were conducted in compliance with the guidelines for the care and use of laboratory animals and were approved by the Institutional Animal Care and Use Committee (IACUC).
Wild animals	No wild animals were used.
Reporting on sex	The findings not only apply to one sex or gender. And sex or gender- based information were not collected in this study.
Field-collected samples	No field-collected samples were used.
Ethics oversight	Animal experiments were conducted in accordance with the laboratory guidelines for animal care and the protocols were approved by the Institutional Animal Care and Use Committee of Shenzhen People's Hospital.

Note that full information on the approval of the study protocol must also be provided in the manuscript.
